# Rational design of near‐infrared absorbing organic dyes: Controlling the HOMO–LUMO gap using quantitative molecular orbital theory

**DOI:** 10.1002/jcc.25731

**Published:** 2018-12-04

**Authors:** Ayush K. Narsaria, Jordi Poater, Célia Fonseca Guerra, Andreas W. Ehlers, Koop Lammertsma, F. Matthias Bickelhaupt

**Affiliations:** ^1^ Department of Chemistry and Pharmaceutical Sciences and Amsterdam Center for Multiscale Modeling (ACMM) Vrije Universiteit Amsterdam De Boelelaan 1083, 1081 HV Amsterdam The Netherlands; ^2^ ICREA Barcelona Spain; ^3^ Department of Inorganic and Organic Chemistry and IQTCUB Universitat de Barcelona Barcelona Spain; ^4^ Leiden Institute of Chemistry, Gorlaeus Laboratories Leiden University, Leiden The Netherlands; ^5^ Van't Hoff Institute for Molecular Sciences University of Amsterdam Science Park 904, 1098 XH Amsterdam The Netherlands; ^6^ Department of Chemistry University of Johannesburg Auckland Park, Johannesburg 2006 South Africa; ^7^ Institute of Molecules and Materials Radboud University Heyendaalseweg 135, 6525 AJ Nijmegen The Netherlands

**Keywords:** NIR absorption, charge‐transfer excitations, density functional calculations, design rules, donor–acceptor systems

## Abstract

Principles are presented for the design of functional near‐infrared (NIR) organic dye molecules composed of simple donor (D), spacer (π), and acceptor (A) building blocks in a D‐π‐A fashion. Quantitative Kohn–Sham molecular orbital analysis enables accurate fine‐tuning of the electronic properties of the π‐conjugated aromatic cores by effecting their size, including silaaromatics, adding donor and acceptor substituents, and manipulating the D‐π‐A torsional angle. The trends in HOMO–LUMO gaps of the model dyes correlate with the excitation energies computed with time‐dependent density functional theory at CAMY‐B3LYP. Design principles could be developed from these analyses, which led to a proof‐of‐concept linear D‐π‐A with a strong excited‐state intramolecular charge transfer and a NIR absorption at 879 nm. © 2018 The Authors. Journal of Computational Chemistry published by Wiley Periodicals, Inc.

## Introduction

Near‐infrared (NIR) absorbing organic dyes (650–950 nm) are highly sought after for application as an emitter in tissue imaging^1^, ^2^ and organic electronics,^3^, ^4^, ^5^, ^6^ and as a photosensitizer in organic photovoltaics.^7^, ^8^, ^9^ Their tunability,^10^, ^11^, ^12^, ^13^ synthetical accessibility, and low toxicity^14^ give them an advantage over alternate inorganic materials.^15^, ^16^, ^17^, ^18^, ^19^ However, advancing such organic dyes is hampered by complex molecular arrangements^20^, ^21^, ^22^ and a large π‐scaffold with poor excited state intramolecular charge transfer (ICT) nature.^23^, ^24^ There are very few closed‐shell neutral organic molecules that absorb effectively in the NIR range and simultaneously exhibit charge‐transfer excitation.^25^, ^26^, ^27^ So far, there is no comprehensive rationale to predict the behavior of the dyes based on the constituents from which they are built. Of course, it is well‐known that both extending the conjugation length^28^ and introducing donor/acceptor push‐pull effects^29^, ^30^, ^31^, ^32^ cause a redshift of the absorption maxima, but how to tune them in tandem is still missing. *Ab initio* methods can unravel the factors controlling orbital energies, overlaps, HOMO–LUMO gaps, and symmetries and thereby might provide insight into a possible causal relationship between the absorption properties and the dye's tuning parameters.

**Scheme 1:**
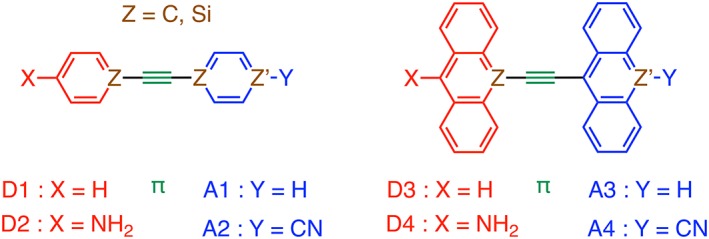
D‐π‐A model systems (D = donor, π = spacer, and A = acceptor). [Color figure can be viewed at wileyonlinelibrary.com]

Here, we present design principles for the rational construction of small dyes with S_0_–S_1_ absorptions (*E*_0_(S1)) in the NIR. Our computational approach is based on a diverse series of modular models composed from simple donor (D), spacer (π), and acceptor (A) building blocks (Scheme 1). The absorption behavior, oscillator strength, and extent of excited state ICT for the linear D‐π‐A frameworks are presented as are the underlying relationships between the electronic and structural changes of the building blocks by using acenes/heteroacenes (i.e., (sila)benzene and (sila)anthracene), their substituents (X: NH_2_ and Y: CN), and the D‐π‐A internal rotation as tuning parameters. A fragment‐based analysis is used to evaluate how the tuning parameters of the separate building blocks transpire into the linear D‐π‐A molecule. From the obtained insights, we design a NIR absorbing organic dye as a proof‐of‐concept.

## Computational Details

### General procedure

All calculations have been performed using Amsterdam Density Functional 2016 quantum chemistry package developed by Software for Chemistry & Materials.^33^, ^34^, ^35^ Electronic ground‐state geometry optimizations have been performed at GGA BP86^36^ in combination with the TZ2P Slater‐type basis set, and a small frozen core (FC). Scalar‐relativistic effects were accounted for by using the zeroth‐order regular approximation (ZORA).^37^ The effect of solvation in dichloromethane (DCM) was simulated using the conductor‐like screening model (COSMO).^38^ All model systems have fully planar equilibrium geometries which were verified to be true minima by performing analytical vibrational frequency analyses. D2‐π(45°)‐A2 and D2(Si)‐π(45°)‐A2 result from rigid rotation of D relative to A by 45°, starting from the planar equilibrium geometry. Kohn–Sham molecular orbital analyses have been performed at the same level of theory, except for the fact that no FC approximation was used (i.e., all electrons were included in the vibrational treatment).

### Time‐dependent density functional theory (TD‐DFT) computations

Linear response TD‐DFT^39^, ^40^ calculations have been performed using the long‐range separated functional CAMY‐B3LYP^41^ in combination with the TZ2P basis set, no FC, with ZORA scalar‐relativistic effect, and with and without nonequilibrium COSMO (DCM) to simulate the DCM solvent environment. For molecules showing excitations with a charge‐transfer nature, long‐range separated functionals exhibit the correct asymptotic nature and thus can successfully predict excitation energies.^42^, ^43^ In addition, extensive benchmarks have been performed earlier by the groups of Tozer and Kronik where they highlighted the ability of the range‐separated functional CAM‐B3LYP to accurately predict various types of excitations in organic dye molecules, in particular, charge‐transfer excitation.^44^, ^45^ Important for these calculations is the γ parameter, which depends on the length of conjugation,^46^, ^47^, ^48^ and might thus have an effect on the models that vary from (sila)benzene to (sila)anthracene. However, the self‐consistently tuned γ parameter, which we obtained using the long‐range corrected LC‐BLYP functional following the procedure mentioned in Ref. 48, gave for D4(Si)‐π‐A4 in DCM virtually the same *E*_0_(S1) of 1.42 eV as the 1.41 eV at CAMY‐B3LYP. It was therefore decided to use CAMY‐B3LYP as an adequate XC functional for estimating the ICT excitations of the model dyes.

## Results and Discussion

### Size of aromatic core

**Figure 1:**
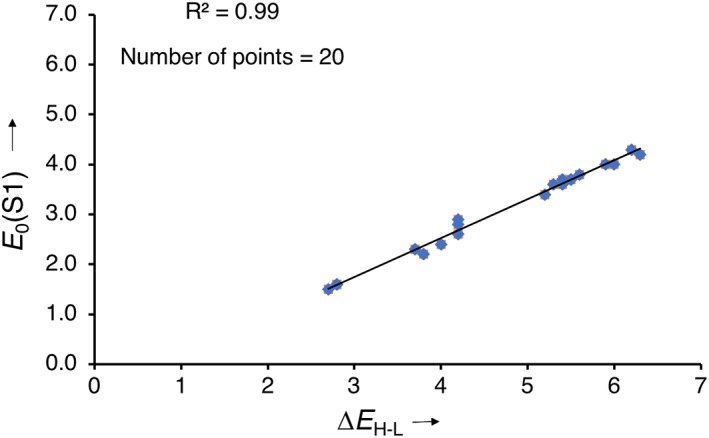
Correlation between HOMO–LUMO gap (Δ*E*_H‐L_) of the D‐π‐A molecule and the lowest singlet excitation energy (*E*_0_(S1)), computed at CAMY‐B3LYP/TZ2P//BP86/TZ2P. [Color figure can be viewed at wileyonlinelibrary.com]

**Figure 2:**
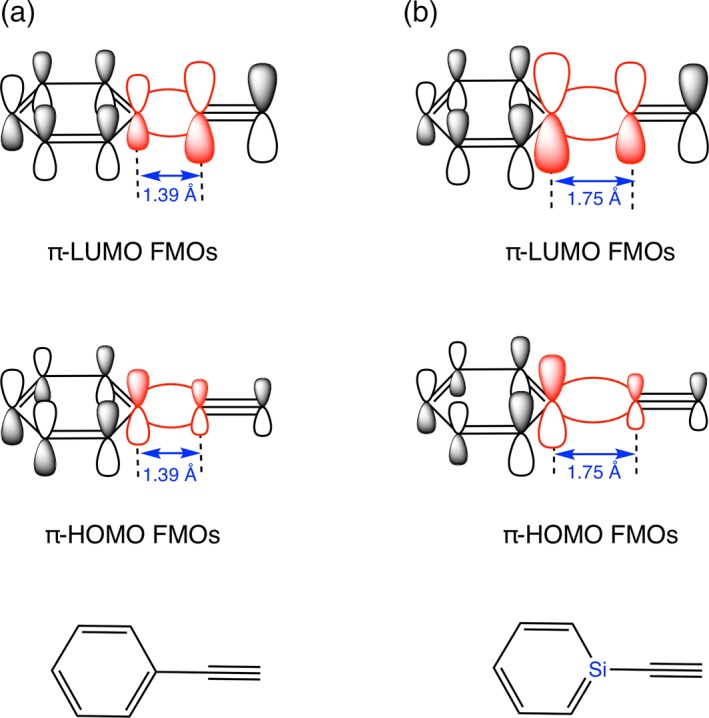
Schematic diagram of the overlap pattern between the π‐HOMO and π‐LUMO FMOs of a) D1‐π and b) D1(Si)‐π. [Color figure can be viewed at wileyonlinelibrary.com]

**Figure 3:**
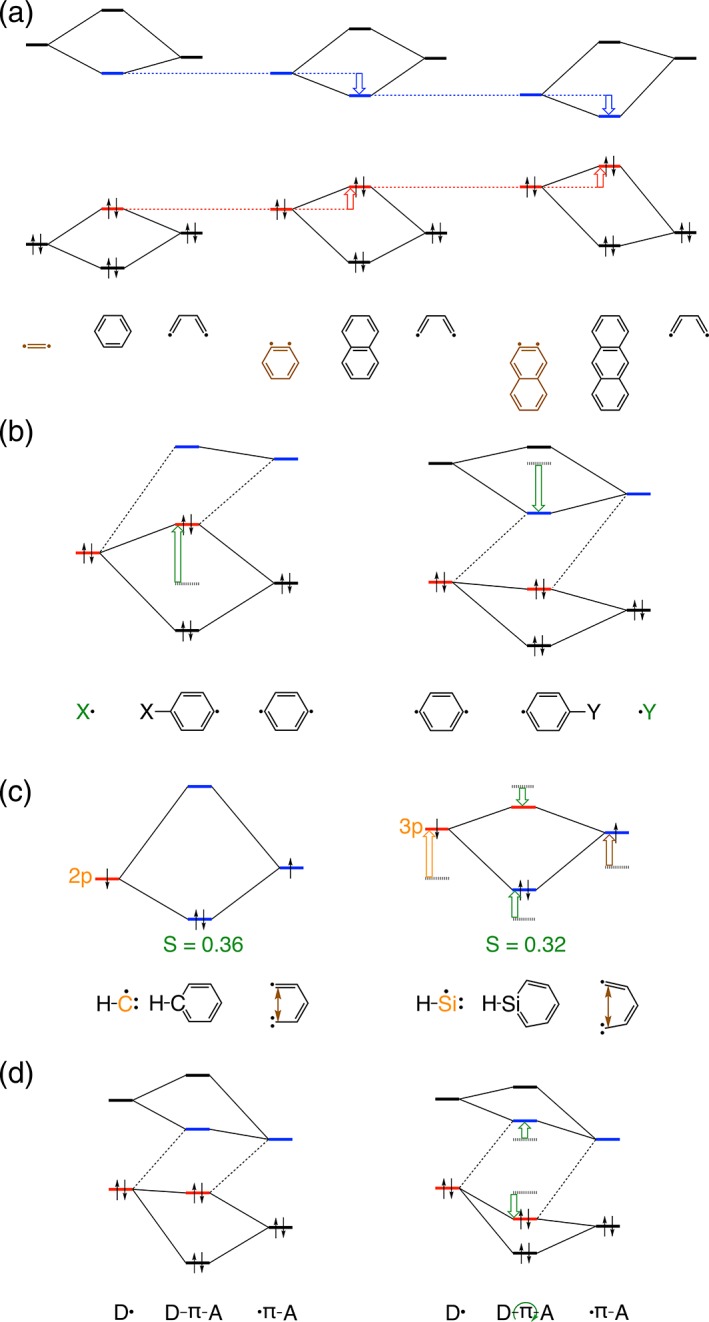
Schematic π orbital‐interaction diagrams (σ electron‐pair bonding is not shown for clarity) based on quantitative KS‐MO analysis highlighting the effect on the HOMO–LUMO gap of: a) increasing the π‐conjugated core size; b) substituents X and Y; c) C‐ or Si‐substitution in the core; and d) internal rotation. Open thick arrows indicate the stabilization or destabilization of MOs relative to parent FMOs (a, b, d) or of silabenzene (F)MOs relative to benzene (F)MOs (c). [Color figure can be viewed at wileyonlinelibrary.com]

**Figure 4:**
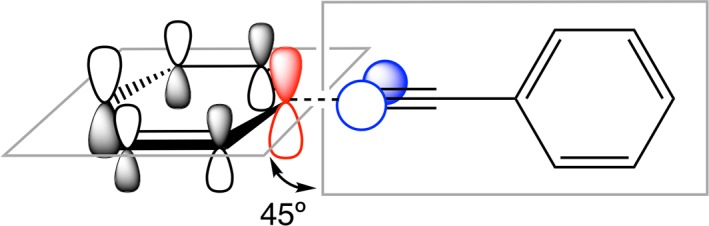
Schematic diagram of the overlap between π‐FMOs in non‐planar D‐π‐A configurations. [Color figure can be viewed at wileyonlinelibrary.com]

**Figure 5:**
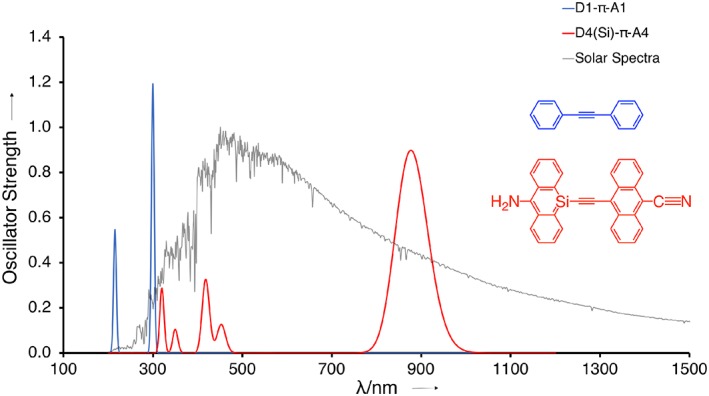
UV/VIS Absorption spectra of two model dyes in DCM solution, computed at COSMO‐CAMY‐B3LYP/TZ2P and overlaid on the measured solar irradiance spectrum above atmosphere.^51^ [Color figure can be viewed at wileyonlinelibrary.com]

The results, summarized in Table 1 and visualized in Figures 1, 2, 3, 4, 5, will be analyzed first in terms of the contributions of each of the D, π, and A components upon which a projection is made on how to achieve an absorption for the D‐π‐A model into the NIR. The absorption maximum of the D‐π‐A model is associated with the excitation from the occupied *ϕ*_i_ to the unoccupied *ϕ*_a_ orbitals and thus their energy difference *ε*_a_ − *ε*_i_. The orbital energy gap, which we aim to understand and tune, shows an excellent correlation with the far more accurate excitation energies calculated at linear response TD‐DFT in which it is also the leading term (Fig. 1). The reason for this behavior is that all S_0_–S_1_ excitations are predominantly single‐electron HOMO–LUMO transitions (see orbital composition of *E*_0_(S1) in Table 1). To address the influence of the building blocks on this energy gap (Δ*E*_H‐L_) of the D‐π‐A molecules, we evaluate the HOMO–LUMO energy differences of the fragments which compose them, denoted as Δ*E* ^frag^_H‐L_. All fragments featuring in our analyses (A, π, D and also X, Y, Z; Scheme 1) are radical species with valence electronic configurations that lead to either doublet, triplet, or quartet states for mono‐, bi‐, and tri‐radicals, respectively. See Supporting Information for all further details regarding structural and energy data as well as quantitative MO interaction diagrams. Increasing the size of the core and thus the conjugation length leads to an expected decrease in the Δ*E* ^frag^_H‐L_ (Fig. 3a), which also follows from an MO analysis (see Supporting Information Figs. S1 and S2). Specifically, changing from benzene (D1/A1) to anthracene (D3/A3) causes a reduction of Δ*E* ^frag^_H‐L_ from 5.11 to 2.33 eV.

**Table 1:** Orbital energies and gap, first singlet excitation energy along with its orbital composition, and oscillator strength for D‐π‐A model dyes.^a^

		Gas								DCM
Name	Description	HOMO^b^	LUMO^b^	Δ*E*_H‐L_ b	Δ*E*_H‐L_ c	*E*_0_(S1)^c^	*E*_0_(S1) (nm)^c^	Orbital composition of *E*_0_(S1) (%)^c^	*f* c	*E*_0_(S1) (nm)^d^
D1‐π‐A1	Ph—C≡C—Ph	−5.6	−2.5	3.1	6.3	4.2	292	95.9	1.0	300
D1(Si)‐π‐A1	Ph(Si)—C≡C—Ph	−5.3	−2.8	2.5	5.5	3.7	335	94.6	0.7	340
D2‐π‐A1	NH_2_—Ph—C≡C—Ph	−4.8	−2.1	2.7	6.0	4.0	310	95.6	1.1	312
D1‐π‐A2	Ph—C≡C—Ph—CN	−6.0	−3.3	2.7	5.9	4.0	310	93.6	1.2	323
D2‐π‐A2	NH_2_—Ph—C≡C—Ph—CN	−5.2	−2.9	2.3	5.3	3.6	344	95.1	1.3	357^e^
D2(Si)‐π‐A2	NH_2_—Ph(Si)—C≡C—Ph—CN	−4.7	−3.2	1.5	4.2	2.8	443	94.2	0.8	470
D2‐π‐(Si)A2	NH_2_—Ph—C≡C—(Si)Ph—CN	−5.4	−3.0	2.4	5.4	3.6	344	91.8	1.2	361
D2‐π(45°)‐A2	NH_2_—Ph—C≡C(45°)—Ph—CN	−5.2	−2.8	2.4	5.4	3.7	335	94.6	0.7	354
D2(Si)‐π(45°)‐A2	NH_2_—Ph(Si)—C≡C(45°)—Ph—CN	−4.8	−3.2	1.6	4.2	2.9	428	95.8	0.4	477
D3‐π‐A3	Ant—C≡C—Ant	−4.9	−3.3	1.6	4.2	2.6	477	95.6	0.6	497
D4‐π‐A4	NH_2_—Ant—C≡C—Ant—CN	−4.7	−3.5	1.2	3.7	2.3	539	97.0	0.8	596
D4(Si)‐π‐A4	NH_2_—Ant(Si)—C≡C—Ant—CN	−4.2	−3.6	0.6	2.8	1.6	775	94.8	0.7	879
D1‐π‐A1(N)	Ph—C≡C—Ph(N)	−5.9	−2.9	3.0	6.2	4.3	288	94.5	0.9	302
D1‐π‐A1(P)	Ph—C≡C—Ph(P)	−5.6	−3.0	2.6	5.6	3.8	326	95.3	1.0	338
D1(Ge)‐π‐A1	Ph(Ge)—C≡C—Ph	−5.3	−2.8	2.5	5.5	3.7	335	94.2	0.7	342
D1(Sn)‐π‐A1	Ph(Sn)—C≡C—Ph	−5.0	−2.7	2.3	5.2	3.4	365	94.2	0.6	373
D4‐π‐A3(N)	NH_2_—Ant—C≡C—Ant(N)	−4.7	−3.3	1.4	4.0	2.4	509	95.1	0.7	553
D4‐π‐A3(P)	NH_2_—Ant—C≡C—Ant(P)	−4.5	−3.3	1.2	3.8	2.2	551	95.8	0.7	601
D4(Ge)‐π‐A4	NH_2_—Ant(Ge)—C≡C—Ant—CN	−4.3	−3.6	0.7	2.8	1.6	775	95.1	0.7	862
D4(Sn)‐π‐A4	NH_2_—Ant(Sn)—C≡C—Ant—CN	−4.1	−3.6	0.5	2.7	1.5	810	95.4	0.6	963

a Energies (in eV, unless stated otherwise).

b Computed at BP86/TZ2P.

c Computed at CAMY‐B3LYP/TZ2P.

d Computed at CAMY‐B3LYP/TZ2P in DCM simulated using the non‐equilibrium COSMO solvation model.

e The calculated value of the CT excitation matches well with the experimentally obtained absorption maximum of 343 nm in DCM.^50^

### X and Y substituents at D and A groups

Introducing a π‐donating NH_2_ (X in Scheme 1) substituent on the benzene ring (D2) increases its HOMO energy (C_6_H_4π,HOMO_), whereas a π‐accepting CN (Y in Scheme 1) substituent (A2) lowers its LUMO energy (C_6_H_4π*,LUMO_), resulting in both cases in a decrease of Δ*E* ^frag^_H‐L_ (Fig. 3b) in qualitative agreement with recent studies.^52^, ^53^ The HOMO arises from the antibonding combination of the occupied D1_π,HOMO_ and NH_2π,HOMO_ fragment orbitals, whereas the LUMO originates from the bonding combination of the unoccupied A1_π*,LUMO_ and CN_π*, LUMO_ fragment orbitals. Therefore, increasing the overlap between the non‐orthogonal FMOs destabilizes the HOMO and stabilizes the LUMO as in D2 and A2, respectively. For example, the NH_2_ group reduces Δ*E* ^frag^_H‐L_ for benzene by 1.4 eV and the CN group causes a reduction by 0.7 eV. This reduction in the energy gap is mediated by the high‐lying lone‐pair orbital NH_2π,HOMO_ and the low‐lying CN_π*,LUMO_ along with the favorable orbital overlap of the π FMOs belonging to the in‐plane substituent with the π FMOs of the aromatic rings. A further decrease results on adding the acetylenic (π) spacer due to extended π‐conjugation facilitated by the coplanarity of its π and π* orbitals with the phenyl rings of the D and A groups. For example, such para‐substitution of aniline causes a reduction of Δ*E* ^frag^_H‐L_ from 3.72 to 3.27 eV. The origin for this effect lies in the relatively low‐lying π_π*,LUMO_ of the acetylenic unit (*ε* = −1.78 eV) which admixes in a bonding fashion with the higher lying NH_2π*,LUMO_ of the aniline fragment (*ε* = −0.88 eV). Experimental studies on the effects of substituting X/Y on diphenylacetylene (c.f., D1‐π‐A1), which exhibits a large fluorescence quantum yield (*Φ*_F_ = 0.50) at low temperature implying good emission properties as well,^54^ have also shown that increasing the donor–acceptor strength causes an increase in the absorption wavelength maximum and hyperpolarizability.^50^, ^54^, ^55^, ^56^, ^57^

### Heteroatom substitution in aromatic core

Another strategy is to introduce a heteroatom into the π‐conjugated core, for which we choose silicon (Z in Scheme 1) as sila‐aromatics have been shown to be realistic synthetic targets.^58^, ^59^, ^60^, ^61^, ^62^, ^63^, ^64^ Our analyses reveal two electronic mechanisms at work that are responsible for the decrease of Δ*E* ^frag^_H‐L_ upon introducing Si into the benzene core (Fig. 3c). (1) The large diffuse character of Si 3p orbitals leads to less effective 〈3p|2p〉 atomic orbital overlap (0.36 and 0.32 in case of C—C and Si—C overlaps, respectively), which is reciprocated in the reduction of energy between the in‐phase (HOMO) and out‐of‐phase (LUMO) molecular orbitals. (2) The large effective size of Si causes a deformation of the conjugated ring, which destabilizes the C_5_H_5π,SOMO_ because this Fragment Molecular Orbital (FMO) has bonding character with the terminal C atoms. This, in turn, raises the HOMO of the resulting six‐membered aromatic core as a result of the bonding combination of the moderately higher SOMO energies of both C_5_H_5π,SOMO_ and the silicon 3*p*_π,SOMO_ of the SiH fragment in silabenzene.

These findings already explain the trends reported in earlier works.^25^, ^49^, ^65^ But there is more. For example, the amplitude of the HOMO and LUMO at the more electropositive Si in the 1‐silaphenyl fragment (D(Si)) appears to be larger than at the corresponding C atom in the phenyl donor fragment D. This means that 1‐silaphenyl donor fragments have a favorable overlap with the π FMOs of the acceptor, π‐A (*vide infra*, Fig. 2).

To probe the generality of our model, we explored potential dyes involving yet other heterosubstituted (E) π‐conjugated cores (at Z, Z′ positions in Scheme 1) that should lower the acceptor's LUMO and raise the donor's HOMO to provide the desired redshift. Indeed, introducing germanium (D1(Ge)‐π‐A1) in the donor and phosphorus (D1‐π‐A1(P)) in the acceptor leads to a smaller *E*_0_(S1) as compared to the parent. Our (D1(E)‐π‐A1) model thus predicts smaller HOMO–LUMO gaps and correspondingly a larger redshift on moving (E) from C‐ to Si/Ge‐ to Sn‐containing acenes (Table 1). A similar although not identical effect is achieved upon introducing nitrogen (D1‐π‐A1(N)) in the acceptor. Note, however, that phosphorus is more similar to silicon than nitrogen in the sense that phosphorus substitution lowers the LUMO energy without sacrificing the HOMO energy, while nitrogen substitution lowers both HOMO and LUMO energies (Table 1). As a result, the efficacy in lowering the HOMO–LUMO gap increases along N, P, and Si.

### Relationship between the tuning parameters

The next step is to verify whether the outlined tuning principles work in an additive manner and whether there actually exists a causal relationship between Δ*E* ^frag^_H‐L_ and Δ*E*_H‐L_. In other words, if the HOMO–LUMO gap is reduced for each of the functional fragments (D, π, A), does this also lead to maximum reduction of the HOMO–LUMO gap of the overall D‐π‐A molecule (Δ*E*_H‐L_, Table 1)? The answer is affirmative.

The targeted minimization of the HOMO–LUMO gap of the overall system is achieved on maximizing the <D_π,HOMO_|A_π,HOMO_> and <D_π*,LUMO_|A_π*,LUMO_> overlaps. For the (sila)phenyl‐containing model systems, the lowest Δ*E*_H‐L_ with effective ICT excitation was found for planar D2(Si)‐π‐A2, which is redshifted by a sizeable Δ*E*_0_(S1) of 1.4 eV from D1‐π‐A1. All the tuning factors work in tandem to minimize the HOMO–LUMO gap. (1) D2(Si) in D2(Si)‐π‐A2 has the highest‐energy D_π,HOMO_ among all the analyzed donor fragments, while the amine substituent and silabenzene also enable a relatively low D_π*,LUMO_ that is well set up to interact with the lower energy π‐A_π*,LUMO_. (2) For D2‐π‐A2 and D2(Si)‐π‐A2, the <D_π,HOMO_|π‐A_π,HOMO_> and <D_π*,LUMO_|π‐A_π*,LUMO_> overlaps (both 0.08) are equal to or slightly larger than for their all‐carbon analogue (0.08 and 0.12, respectively) due to the higher amplitude of D(Si)_π,HOMO/LUMO_ on Si, which compensates for the larger inter‐fragment distance of 1.75 Å in D2(Si)‐‐‐π‐A2 *versus* 1.39 Å in D2‐‐‐π‐A2 (*vide supra*). (3) The spatial distribution of the HOMO and LUMO is localized more on D and A, respectively, for D2(Si)‐π‐A2 than for D1‐π‐A1, leading to a more pronounced ICT excitation of *E*_0_(S1) (Supporting Information Fig. S19). Substitution (Z) in the acceptor (D2‐π‐(Si)A2) leads to an increase of *E*_0_(S1) along with a skewed ICT as the HOMO and LUMO amplitudes are both localized on the acceptor. Therefore, heteroatom substitution of C for Si in the acceptor will have little or no effect.

All D‐π‐A models have planar equilibrium geometries at variance with the twisted nature of biphenyl (D1‐A1 in our nomenclature).^66^ However, internal rotation of D relative to A has a rigid rotational barrier of only 1.8 kcal mol^−1^ for D2(Si)‐π‐A2. Hence, structurally more confining scaffolds or environment effects might readily lead to twisted geometries. Such a low rotational barrier is a direct consequence of having acetylene as the π‐bridge. Rotating the torsional angle of D2(Si)‐π‐A2 from planarity (0°) to twisted (45°) occurs at an energetic penalty of a mere 1.3 kcal mol^−1^ and reduces the π–π overlap from 0.08 to 0.06 for <D_π,HOMO_|π‐A_π,HOMO_> (Fig. 4). It (1) causes a slight increase in *E*_0_(S1) of 0.1 eV (Fig. 3d) and (2) reduced delocalization of the frontier orbitals to give enhanced ICT excitation. Thus, increased torsion is beneficial for enhancing ICT at the expense of the blueshifted *E*_0_(S1).

### Design of NIR dye for harvesting solar energy

With the aim to push the absorption of our model into the NIR region, we increased the π‐conjugated core from benzene to anthracene units. Spectra from time‐resolved fluorescence experiments on 1,2‐Bis(9‐anthryl)acetylene show a broad CT‐like band in polar or semipolar solvents.^67^ This suggests a large change in dipole moment upon excitation which we felt would be magnified by introducing appropriate X‐ and Y‐substituents on and/or including a heteroatom in the aromatic moieties.

Although D2‐π‐A2 has an absorption energy *E*_0_(S1) of 3.6 eV (344 nm), it drops to 2.3 eV (539 nm) for D4‐π‐A4 (Table 1). Si substitution reduces *E*_0_(S1) further to 1.6 eV (775 nm) for D4(Si)‐π‐A4, which is our best model dye with a strong NIR absorption (Fig. 5). In contrast to the all‐carbon case, the absorption spectrum of D4(Si)‐π‐A4 exhibits peaks in the VIS region as well due to local excitations on the anthracene unit, implying absorption over a wider range of the solar spectrum (Fig. 5). Solvation may move the main absorption peak even further into the NIR domain as it would stabilize the charge separation that occurs upon excitation. This was corroborated by COSMO solvent calculations on all model systems, among which D4(Si)‐π‐A4 showed a large redshift of 104 nm in DCM as well as a strong ICT excitation (see Table 1 and Supporting Information Fig. S22). This proof‐of‐concept of harnessing the NIR photons of the sunlight was extended to a more diverse series of heteroatoms (E = N, P, Ge, and Sn) in the polyacene (lower four entries in Table 1). As expected from our analysis of heteroatom substitution, the *E*_0_(S1) decreases systematically as we move (E) from C‐ to Si/Ge‐ to Sn‐ substituted anthracene in D4(E)‐π‐A4. The most pronounced NIR absorption along this series is, as our model predicts, for D4(Sn)‐π‐A4 with an absorption maximum at 963 nm.

## Conclusion

We have quantum‐chemically developed an approach for rationally designing organic D‐π‐A dye molecules with a particular HOMO–LUMO or, more precisely, optical gap. Our approach is based on a modular scheme, in which D, π, and A building blocks of an appropriate electronic nature can be combined. Our approach yields a toolbox of design principles that are based on a physical insight into the causal relationships between the MO electronic structure of the functional building blocks and the resulting optical spectrum of the overall dye molecule. Thus, qualitative rational design and quantitative prediction can be achieved consistently within one approach based on Kohn–Sham MO theory in conjunction with TD‐DFT.

The tuning parameters comprise: (1) the size of the donor (D) and/or acceptor (A) aromatic cores (i.e., benzene and anthracene), (2) their π‐electron pushing and accepting substituents, (3) heteroatom substitution in the aromatic core, and (4) the D‐π‐A internal rotation. We find that the HOMO–LUMO gap of an overall D‐π‐A dye molecule inherits the tuning parameters' effects on the electronic properties of the fragments in a more or less additive manner. This implies that one can design organic dyes by first optimizing the individual functional building blocks and then combine them for amplification to predictable absorption maxima.

The understanding gained can be applied to various other kind of model systems to predict *a priori* the absorption maximum in those dye molecules. The NH_2_—Ant(Si)—C≡C—Ant—CN molecule serves as a proof‐of‐concept. This model system has a computed NIR absorption at 879 nm and exhibits strong excited‐state ICT nature, which is desirable for solar energy capture and conversion.

## Supporting information

Supporting Information
