# Supplementary material for: Rational design of near‐infrared absorbing organic dyes: Controlling the HOMO–LUMO gap using quantitative molecular orbital theory
Source: J Comput Chem. 2018 Dec 4;39(32):2690–6. doi: 10.1002/jcc.25731 (PMC6587560; doi:10.1002/jcc.25731)
Supplement: Supplementary file 1 — Supporting Information [file JCC-39-2690-s001.pdf]

Supporting Information for

Rational Design of Near-Infrared Absorbing Organic Dyes: Controlling the HOMO–LUMO Gap using Quantitative MO Theory

Ayush K. Narsaria,[a] Jordi Poater, [b,c] C. Fonseca Guerra,[a,d] Andreas W. Ehlers,[a,e,f] Koop Lammertsma,*[a,f] and F. Matthias Bickelhaupt*[a,g]

Content

S1. Orbital Interaction Diagrams

Figure S1. Fragment Frontier Molecular Orbital diagram of core benzene.

Figure S2. Fragment Frontier Molecular Orbital diagram of core Anthracene.

Figure S3. Fragment Frontier Molecular Orbital diagram explaining the effect of C.

Figure S4. Fragment Frontier Molecular Orbital diagram explaining the effect of Si.

Figure S5. Fragment Frontier Molecular Orbital diagram of D2 radical.

Figure S6. Fragment Frontier Molecular Orbital diagram of D2(Si) radical.

Figure S7. Fragment Frontier Molecular Orbital diagram of A2 radical.

Figure S8. Fragment Frontier Molecular Orbital diagram of A2(Si) radical.

Figure S9. Fragment Frontier Molecular Orbital diagram of D2-π radical.

Figure S10. Fragment Frontier Molecular Orbital diagram of D2(Si)-π radical.

Figure S11. Fragment Frontier Molecular Orbital diagram of A2-π radical.

Figure S12. Fragment Frontier Molecular Orbital diagram of A2(Si)-π radical.

Figure S13. Fragment Frontier Molecular Orbital diagram of D2-π-A2.

Figure S14. Fragment Frontier Molecular Orbital diagram of D2(Si)-π-A2.

Figure S15. Fragment Frontier Molecular Orbital diagram of D2-π-A2(Si).

Figure S16. Fragment Frontier Molecular Orbital diagram of D2-π-A2.

Figure S17. Fragment Frontier Molecular Orbital diagram of D2(Si)-π-A2.

Figure S18. Fragment Frontier Molecular Orbital diagram of D2-π-A2(Si).

Figure S19. MO density pictures highlighting the spatial separation of frontier MOs.

Figure S20. Fragment Frontier Molecular Orbital diagram of D2-π(45°)-A2.

Figure S21. Fragment Frontier Molecular Orbital diagram of D2(Si)-π(45°)-A2.

Figure S22. MO density pictures highlighting the spatial separation of frontier MOs.

S2. References

S3. Cartesian coordinates of stationary points, frequencies and total bond energies

S1. Orbital Interaction Diagrams

For all the MO diagrams below, in Figures S1 - S22, the overlap between the FMOs involved in the formation of the overall MO has been denoted by pink and the contribution of the FMO to the MO has been denoted by black. Overlap for the ground state charge transfer interaction is enclosed within brackets. The color of the MO signifies the FMO with major contribution. $\Delta E^{frag}_{H\text{-}L}$ or $\Delta E_{H\text{-}L}$ is the HOMO–LUMO gap in case of fragment and whole D-π-A system respectively has been denoted in brown. The diagrams are not to scale but do show the correct relative orbital energy ordering of fragment and overall MOs. All the calculations have been performed in ADF2016.[1-3]

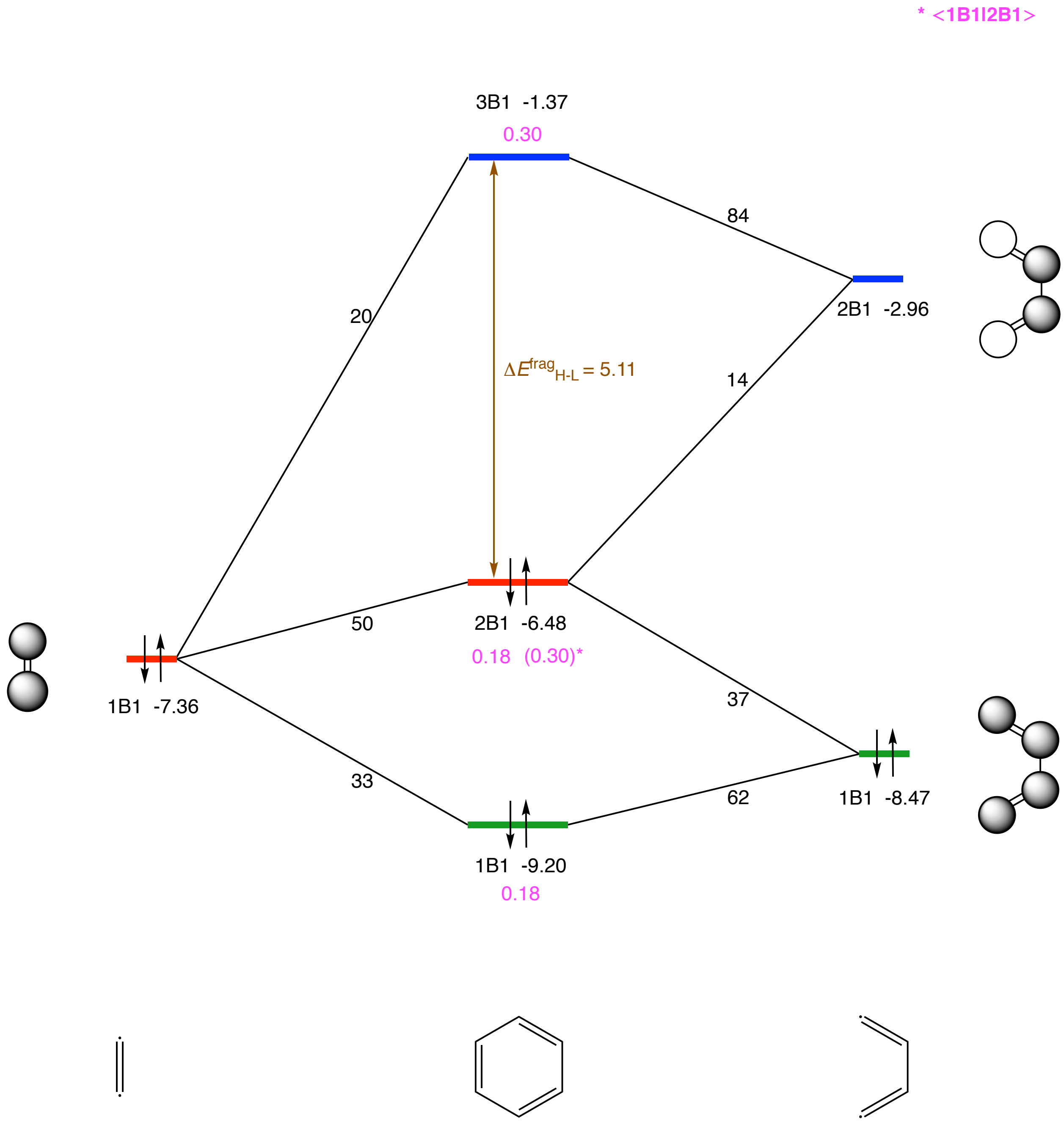

Figure S1. Fragment Frontier Molecular Orbital diagram of core benzene (see text under Section S1, for details).

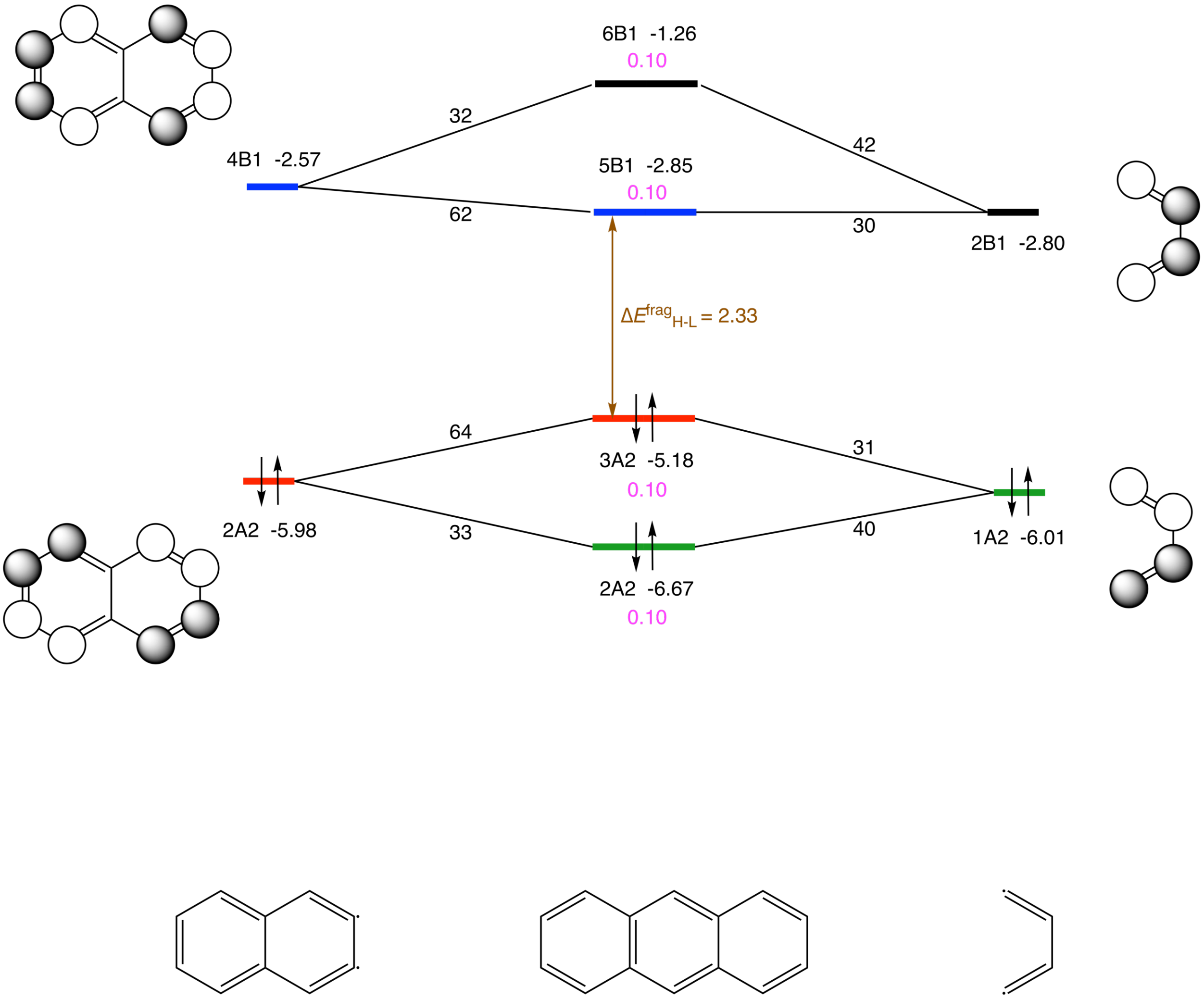

Figure S2. Fragment Frontier Molecular Orbital diagram of core Anthracene (see text under Section S1, for details).

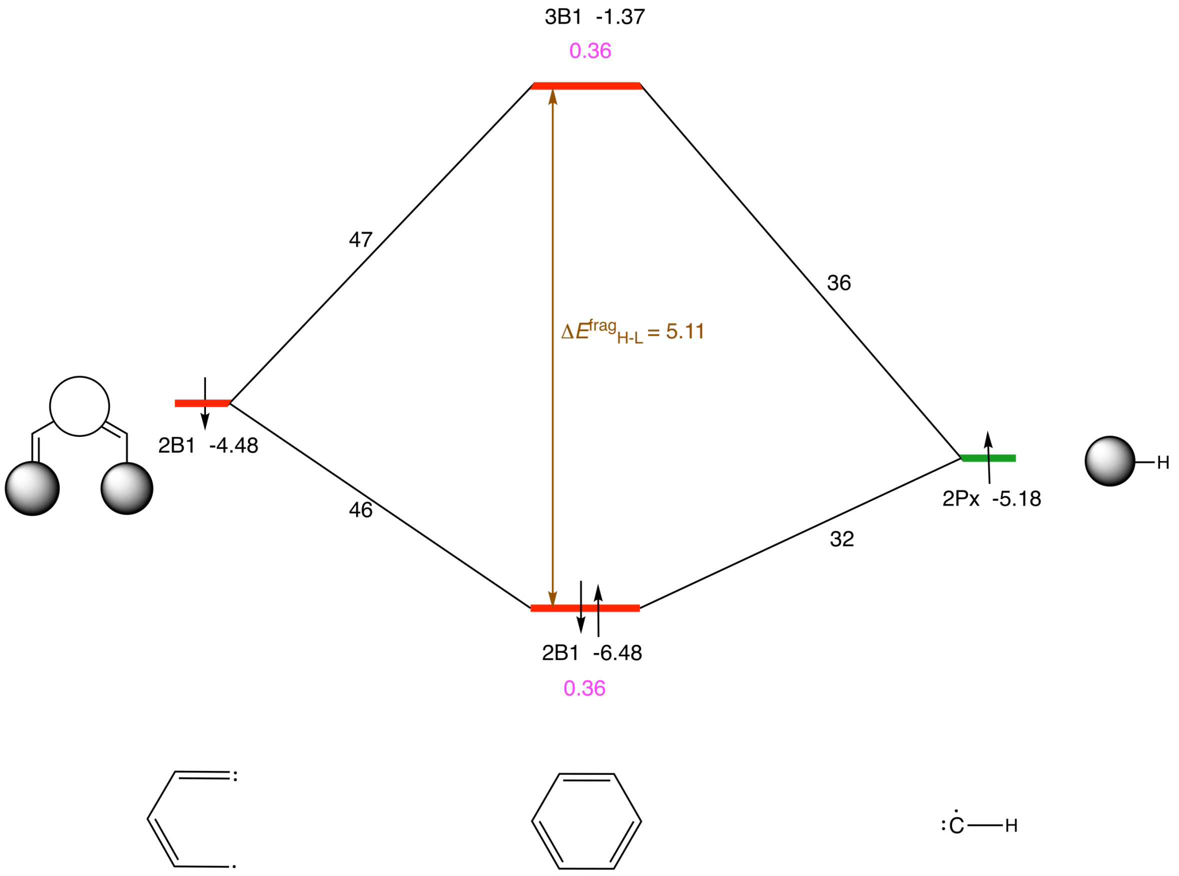

Figure S3. Fragment Frontier Molecular Orbital diagram explaining the effect of C (see text under Section S1, for details).

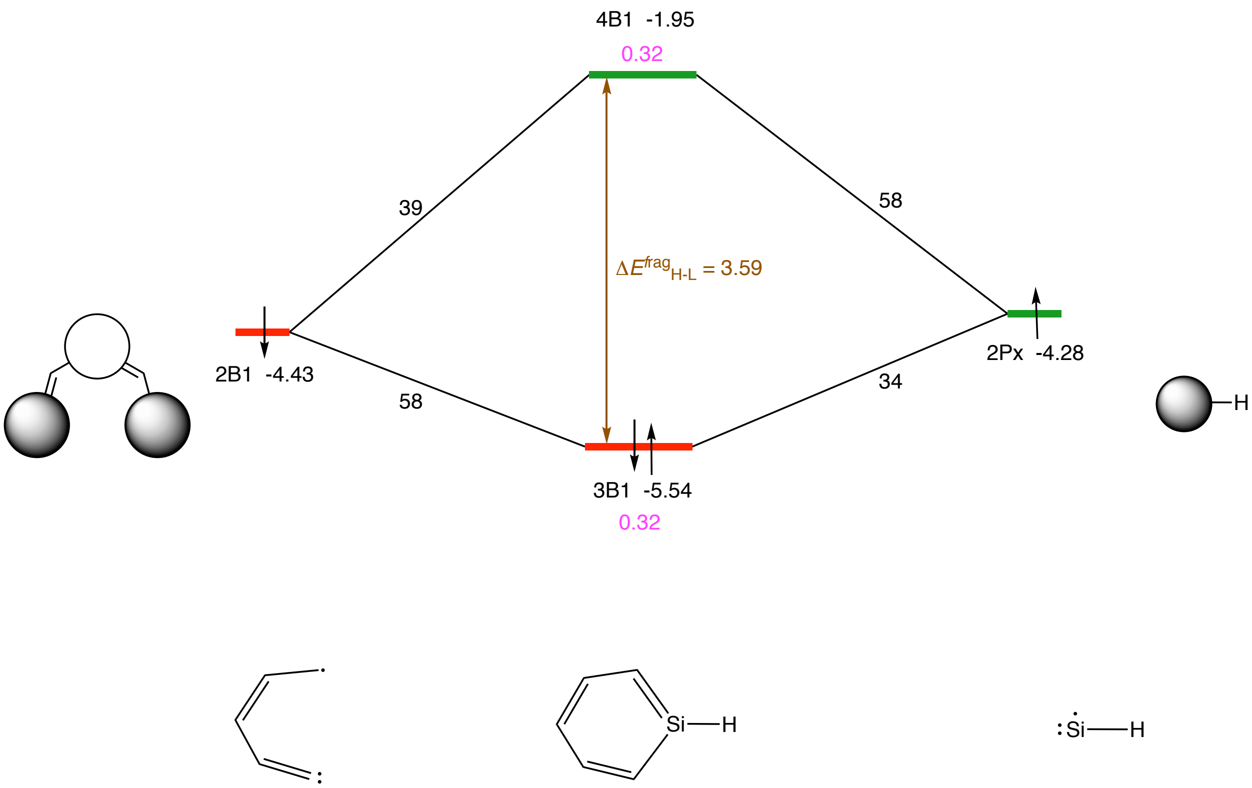

Figure S4. Fragment Frontier Molecular Orbital diagram explaining the effect of Si (see text under Section S1, for details).

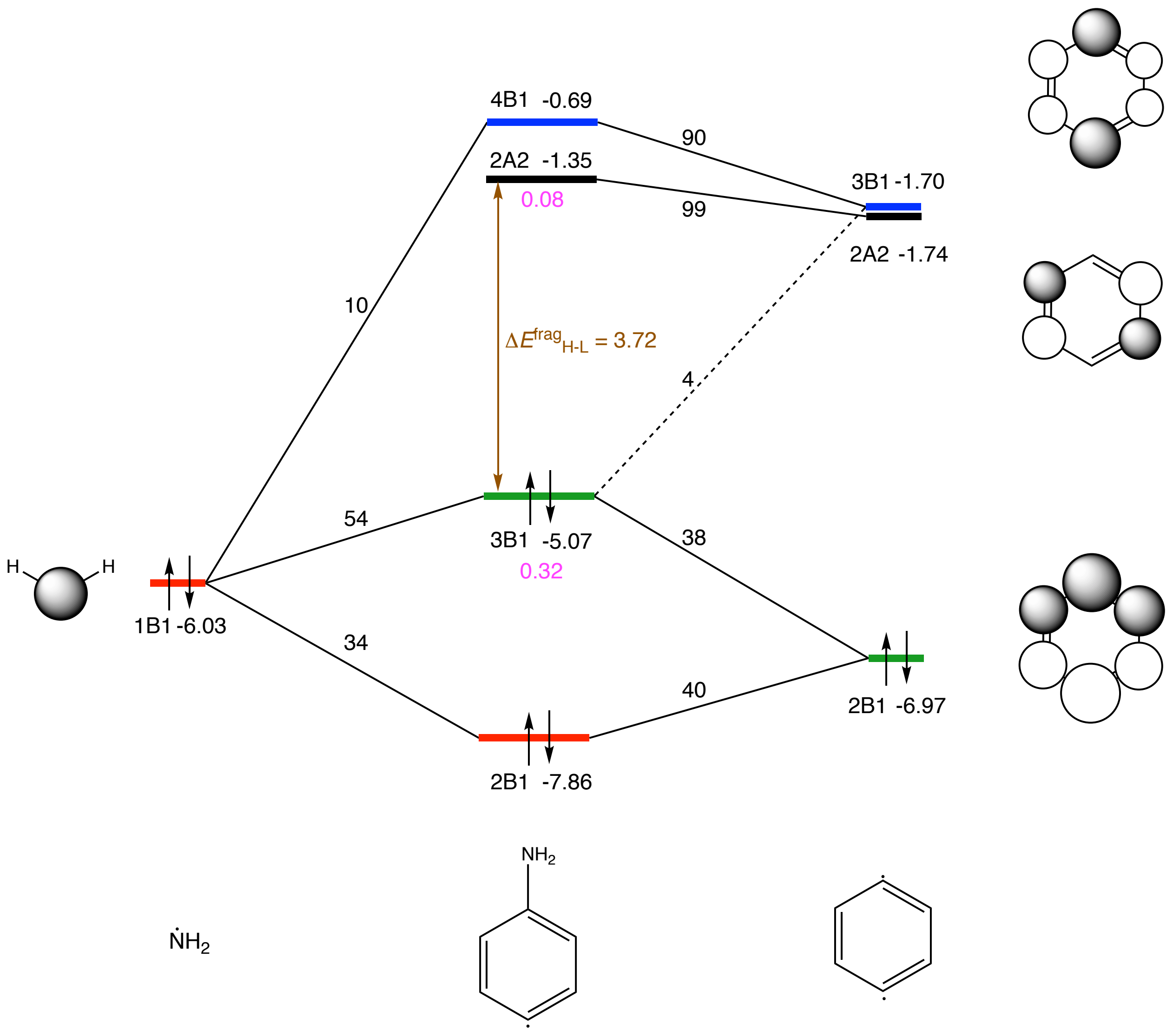

Figure S5. Fragment Frontier Molecular Orbital diagram of D2 radical (see text under Section S1, for details).

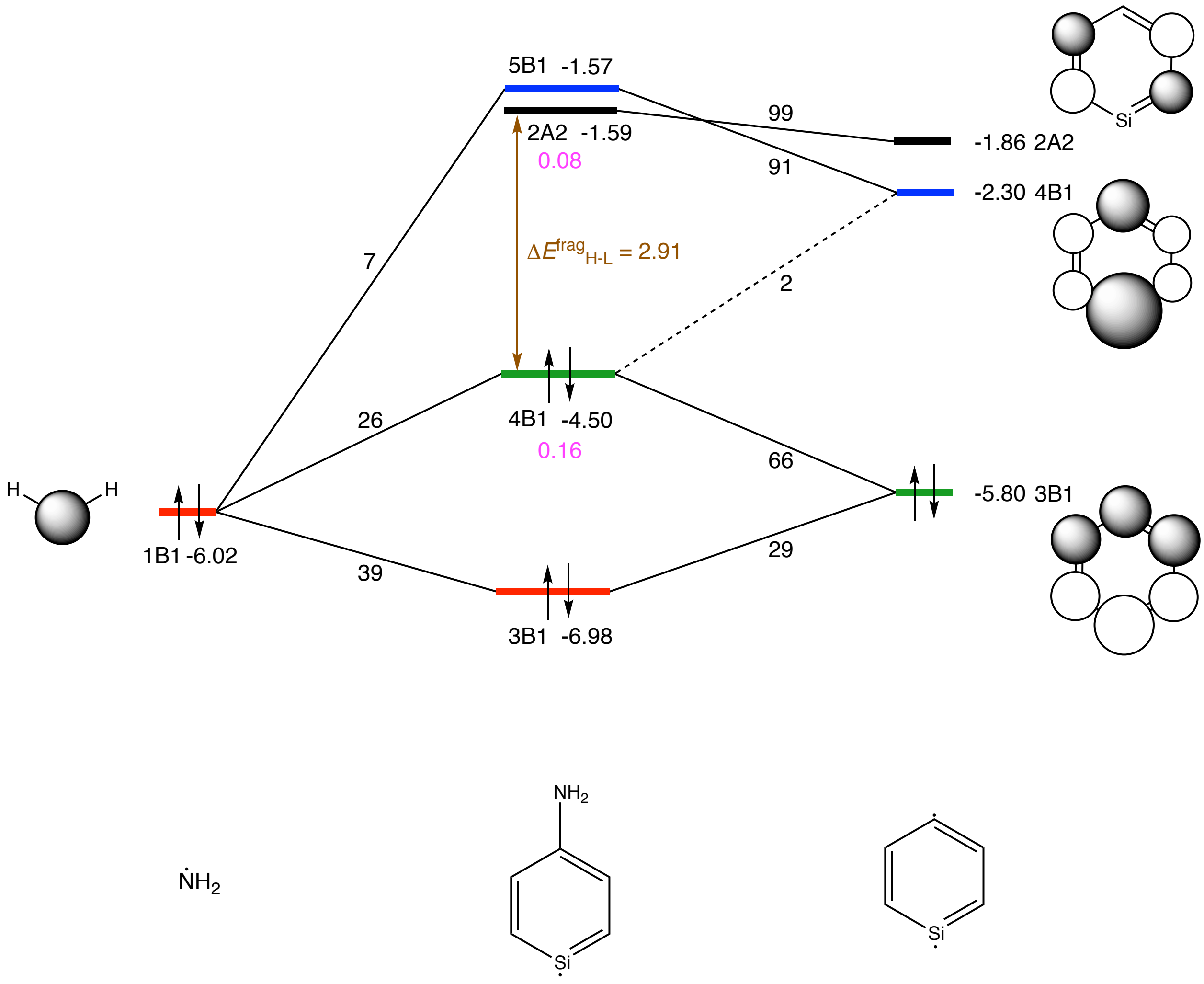

Figure S6. Fragment Frontier Molecular Orbital diagram of D2(Si) radical (see text under Section S1, for details).

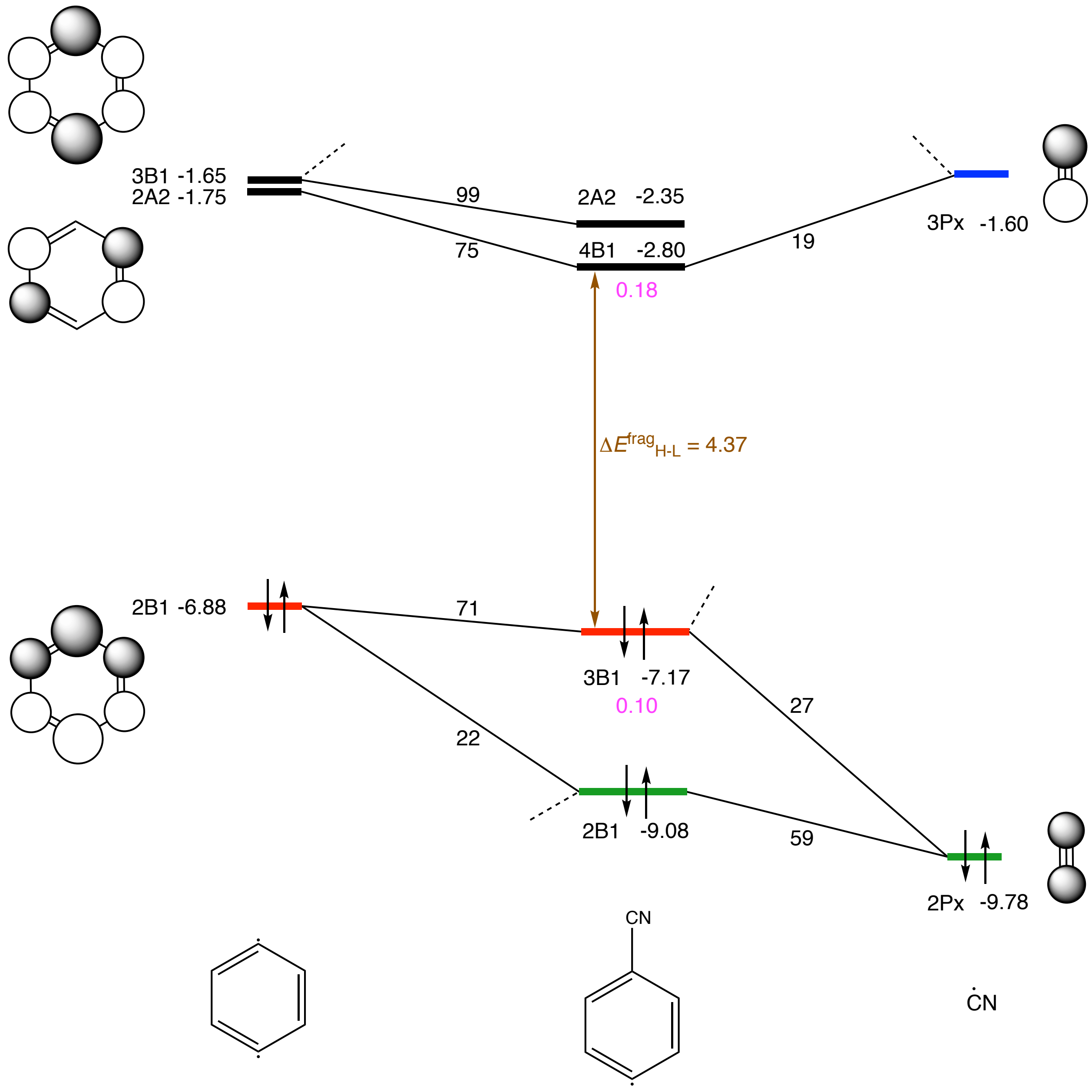

Figure S7. Fragment Frontier Molecular Orbital diagram of A2 radical (see text under Section S1, for details).

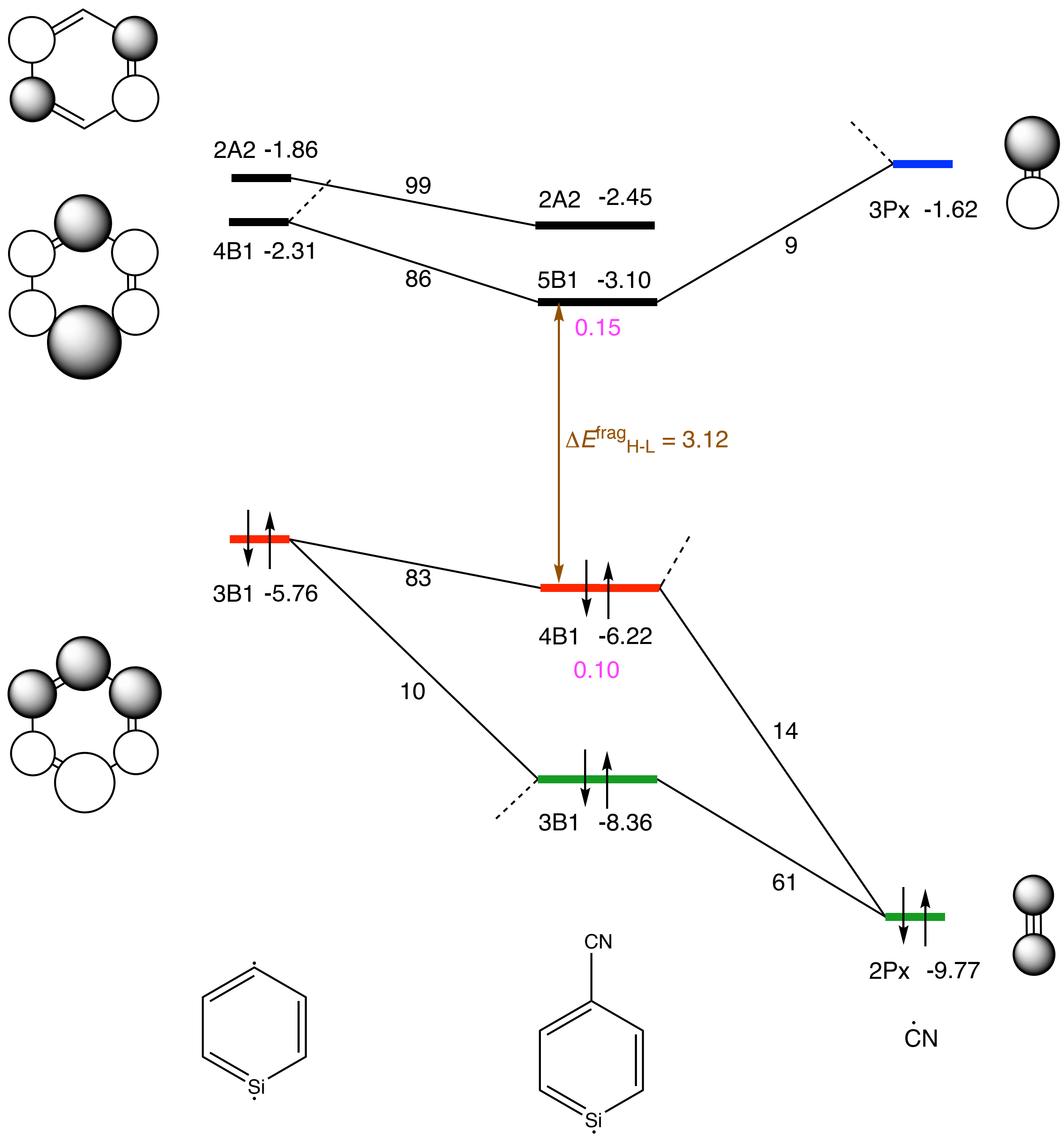

Figure S8. Fragment Frontier Molecular Orbital diagram of A2(Si) radical (see text under Section S1, for details).

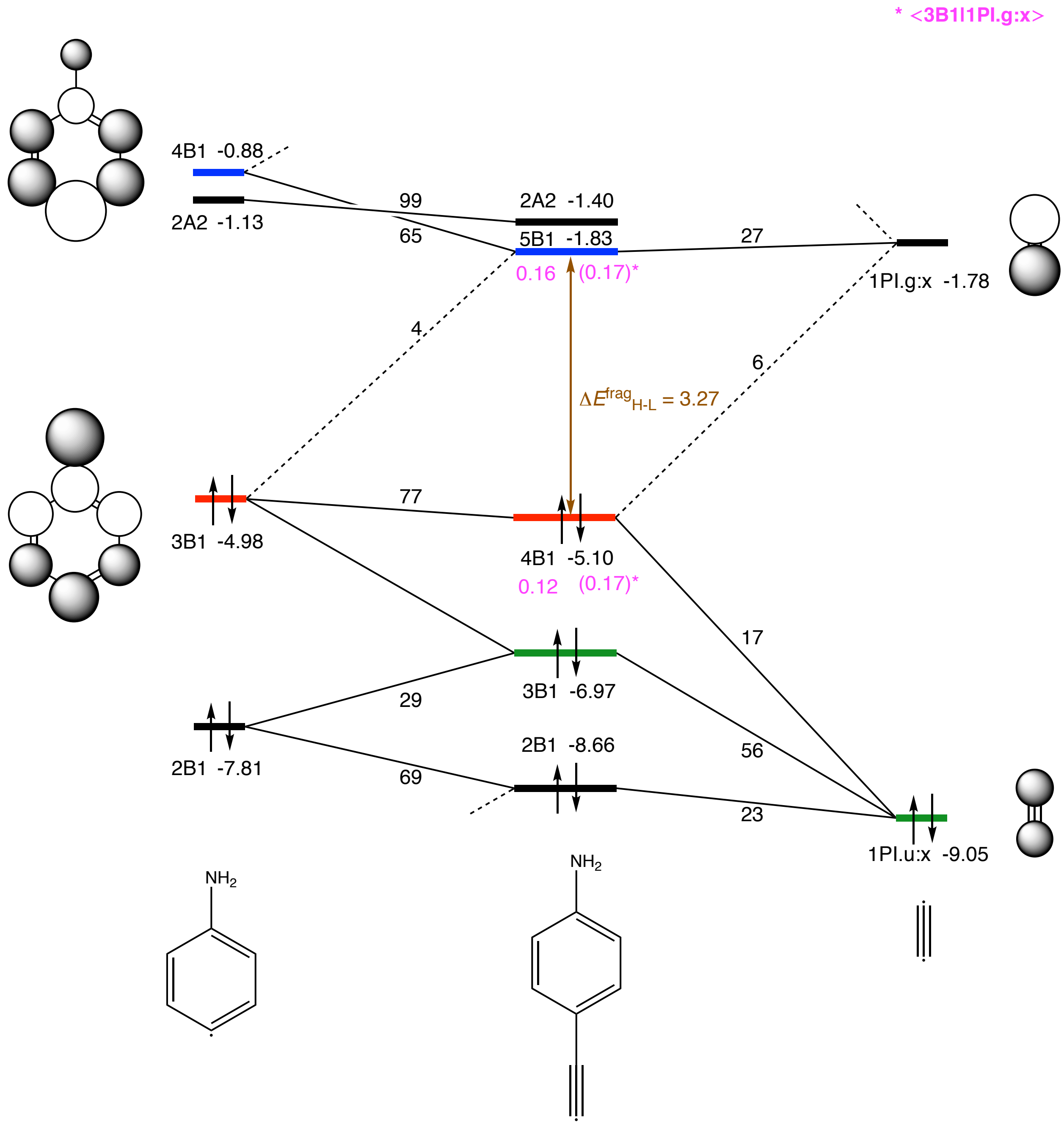

Figure S9. Fragment Frontier Molecular Orbital diagram of D2-π radical (see text under Section S1, for details).

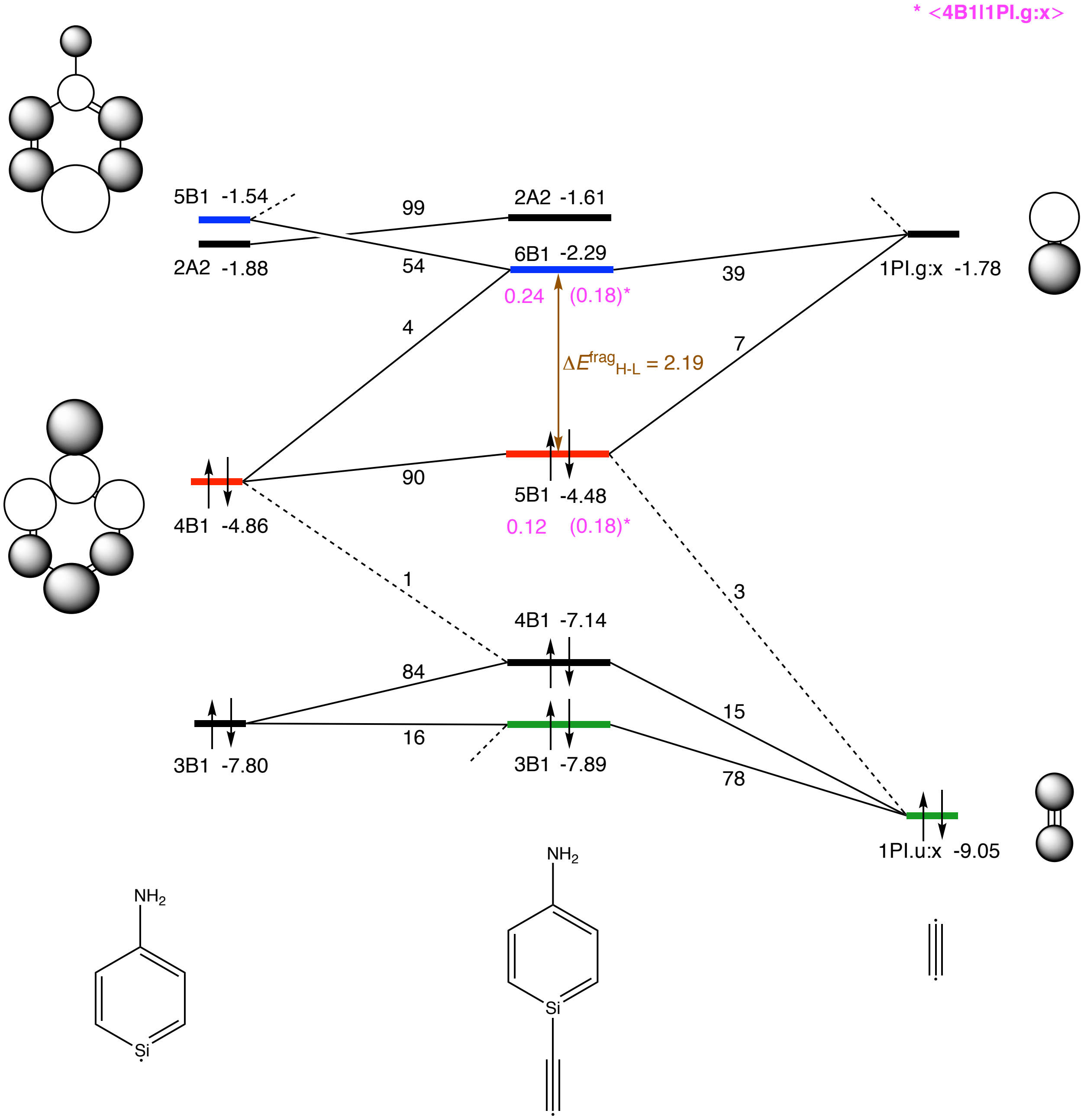

Figure S10. Fragment Frontier Molecular Orbital diagram of D2(Si)-π radical (see text under Section S1, for details).

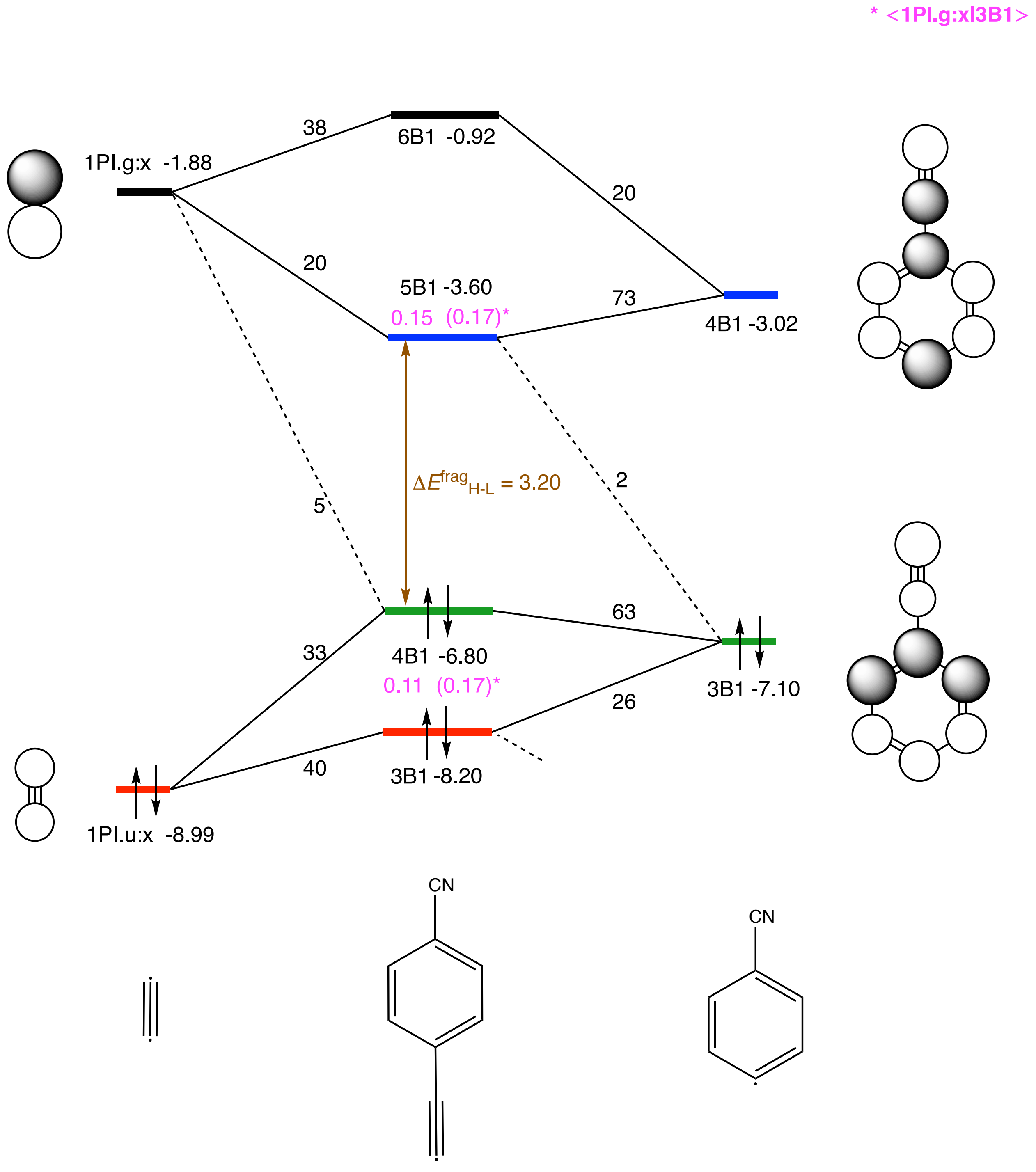

Figure S11. Fragment Frontier Molecular Orbital diagram of A2-π radical (see text under Section S1, for details).

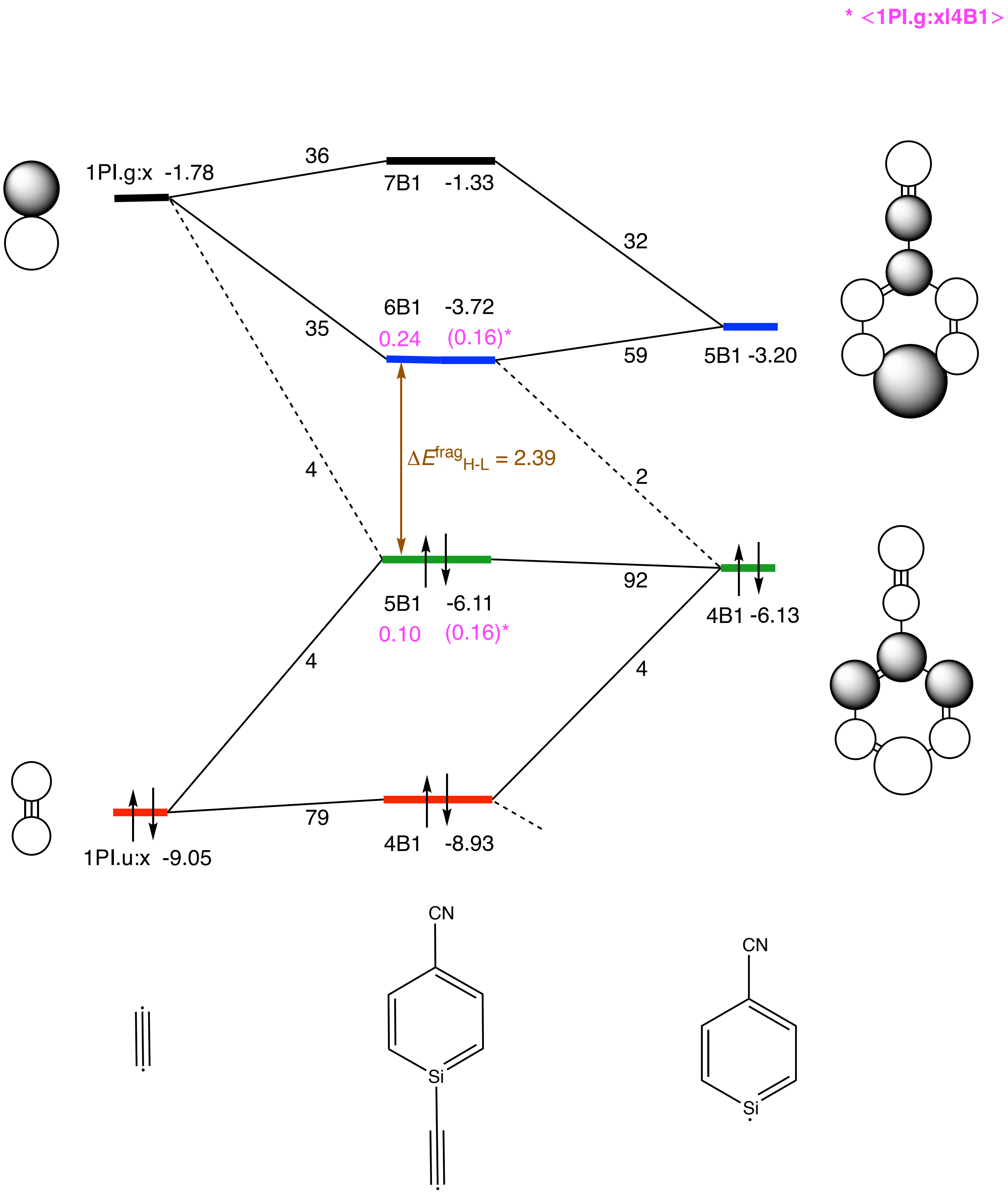

Figure S12. Fragment Frontier Molecular Orbital diagram of A2(Si)-π radical (see text under Section S1, for details).

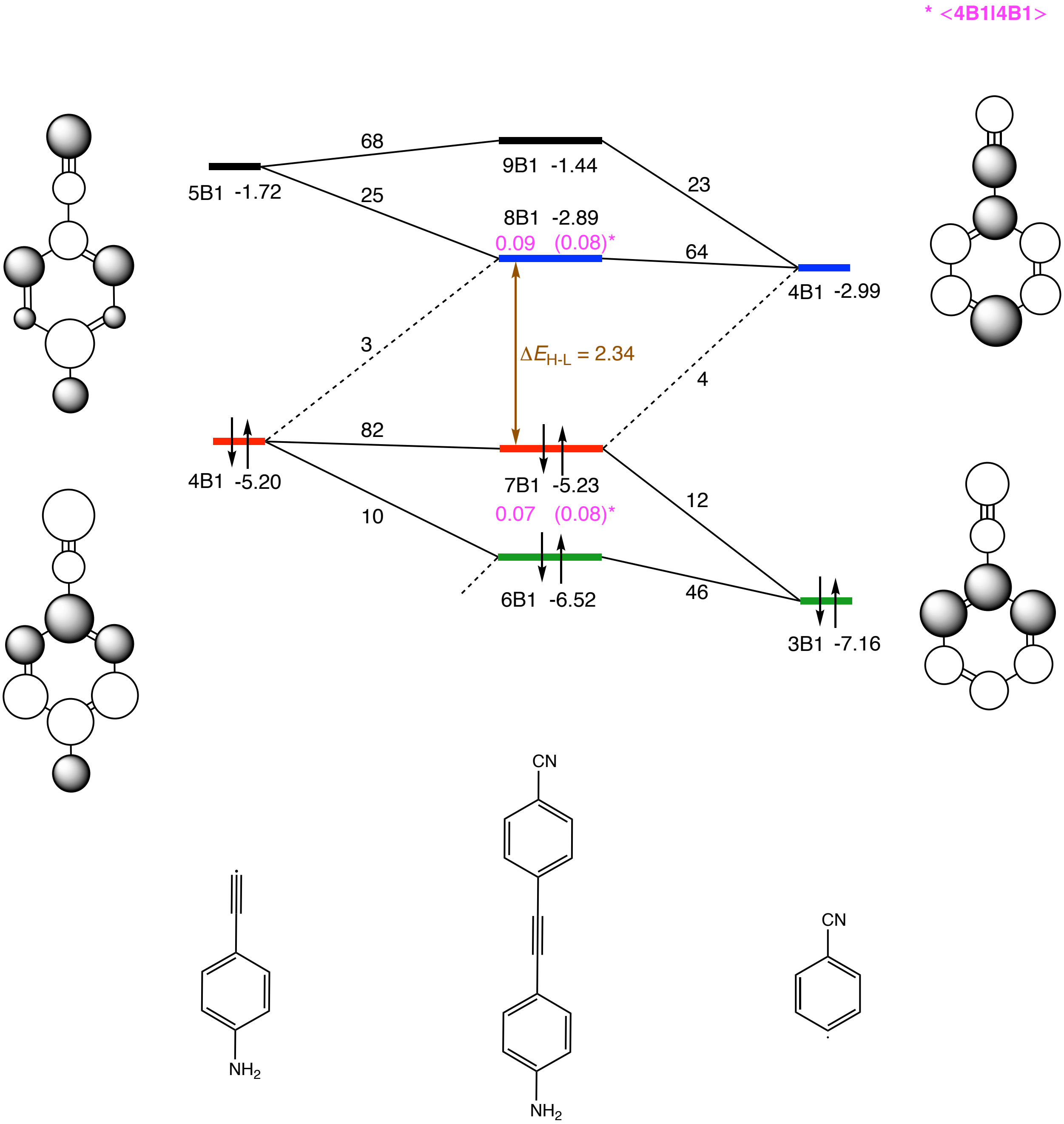

Figure S13. Fragment Frontier Molecular Orbital diagram of D2-π-A2 (see text under Section S1, for details).

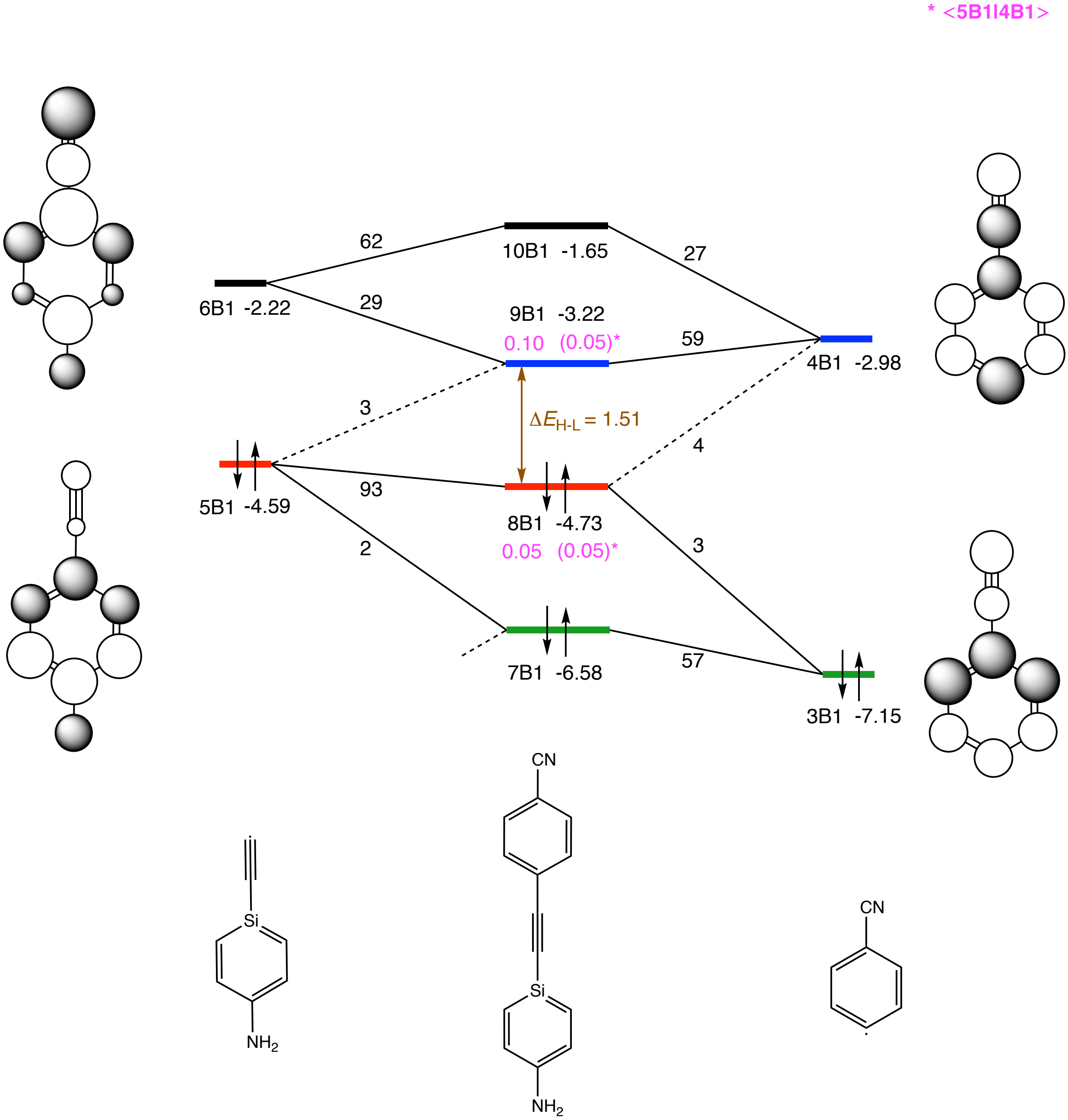

Figure S14. Fragment Frontier Molecular Orbital diagram of D2(Si)-π-A2 (see text under Section S1, for details).

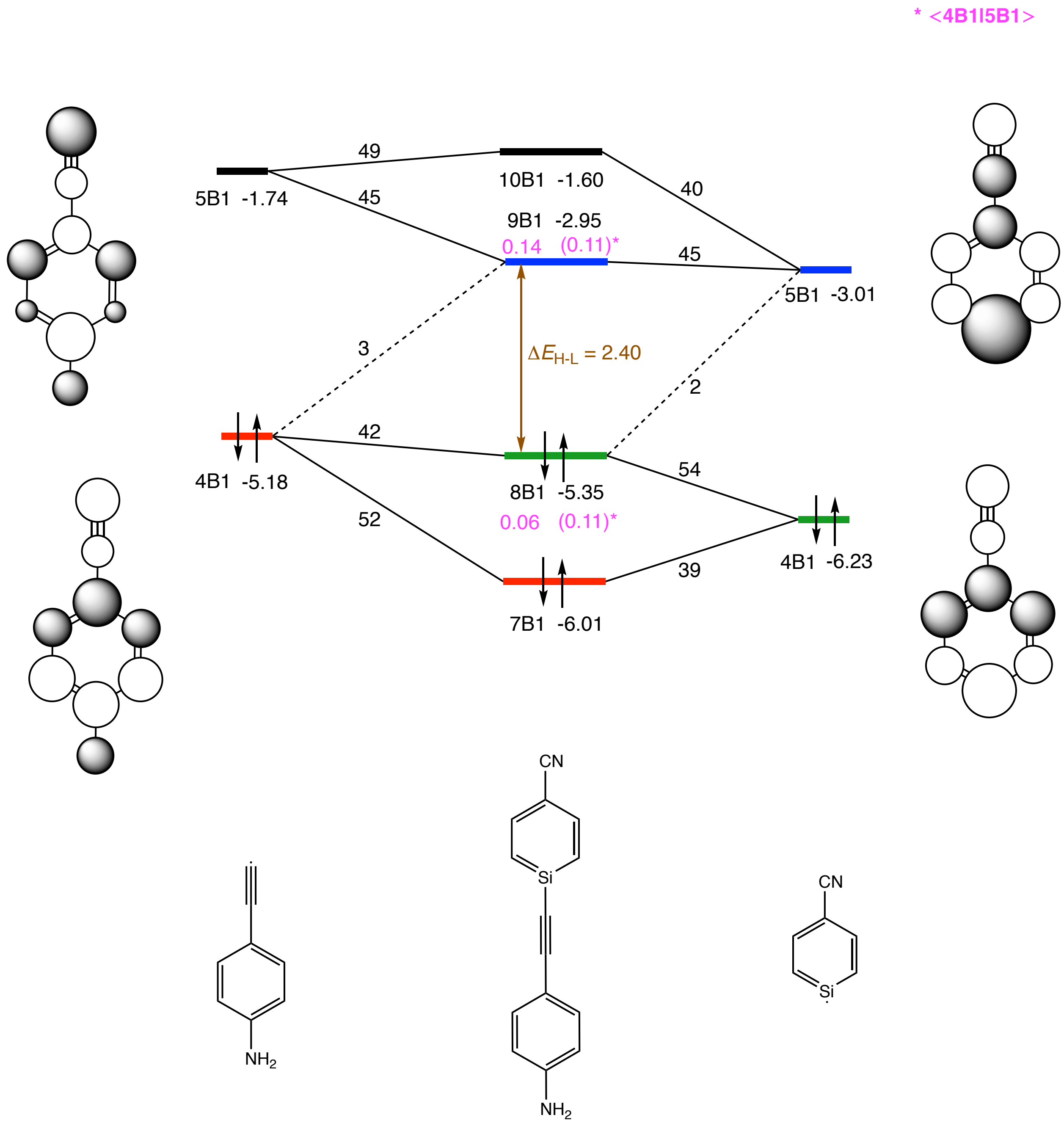

Figure S15. Fragment Frontier Molecular Orbital diagram of D2-π-A2(Si) (see text under Section S1, for details).

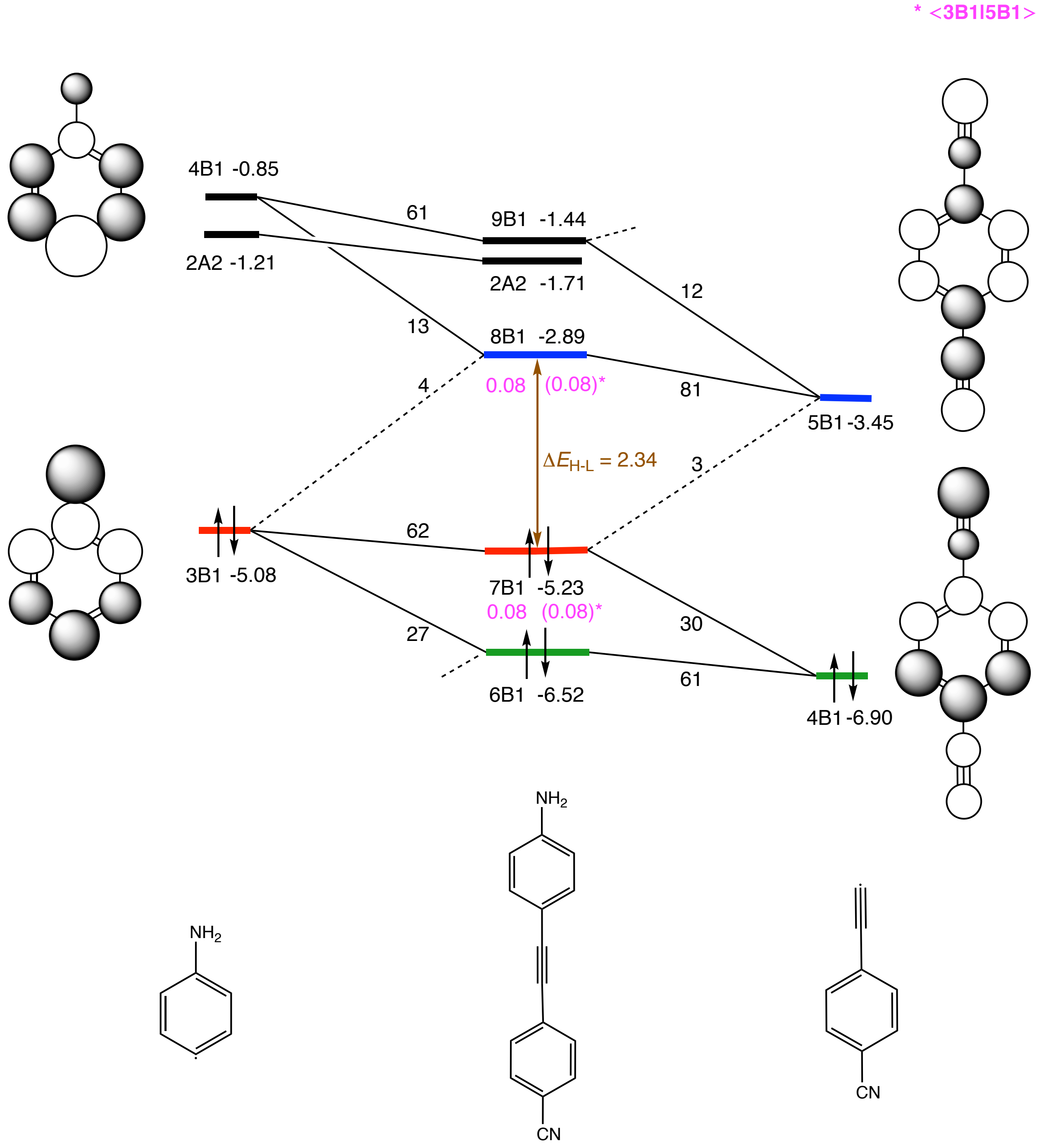

Figure S16. Fragment Frontier Molecular Orbital diagram of D2-π-A2 (see text under Section S1, for details).

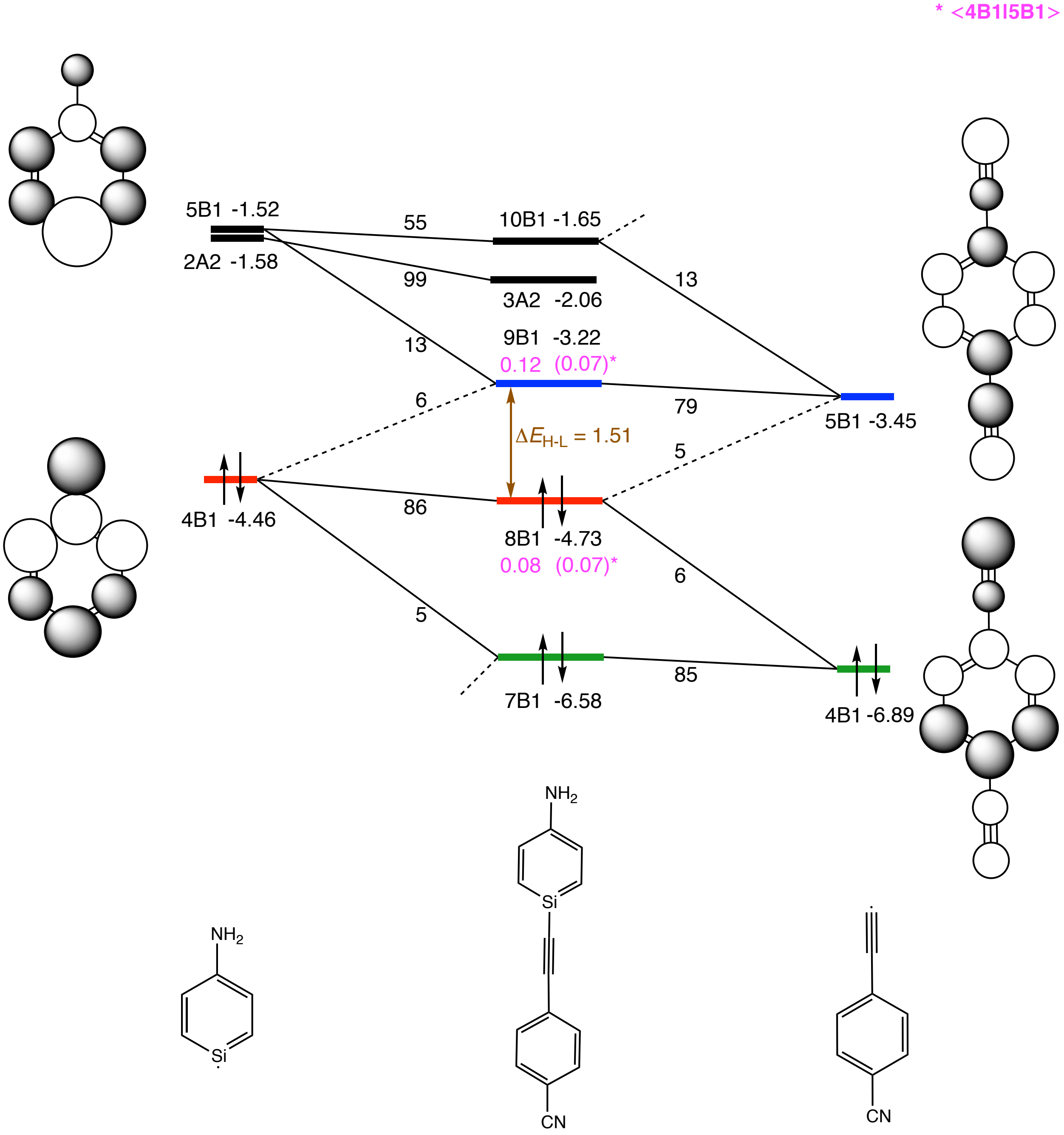

Figure S17. Fragment Frontier Molecular Orbital diagram of D2(Si)-π-A2 (see text under Section S1, for details).

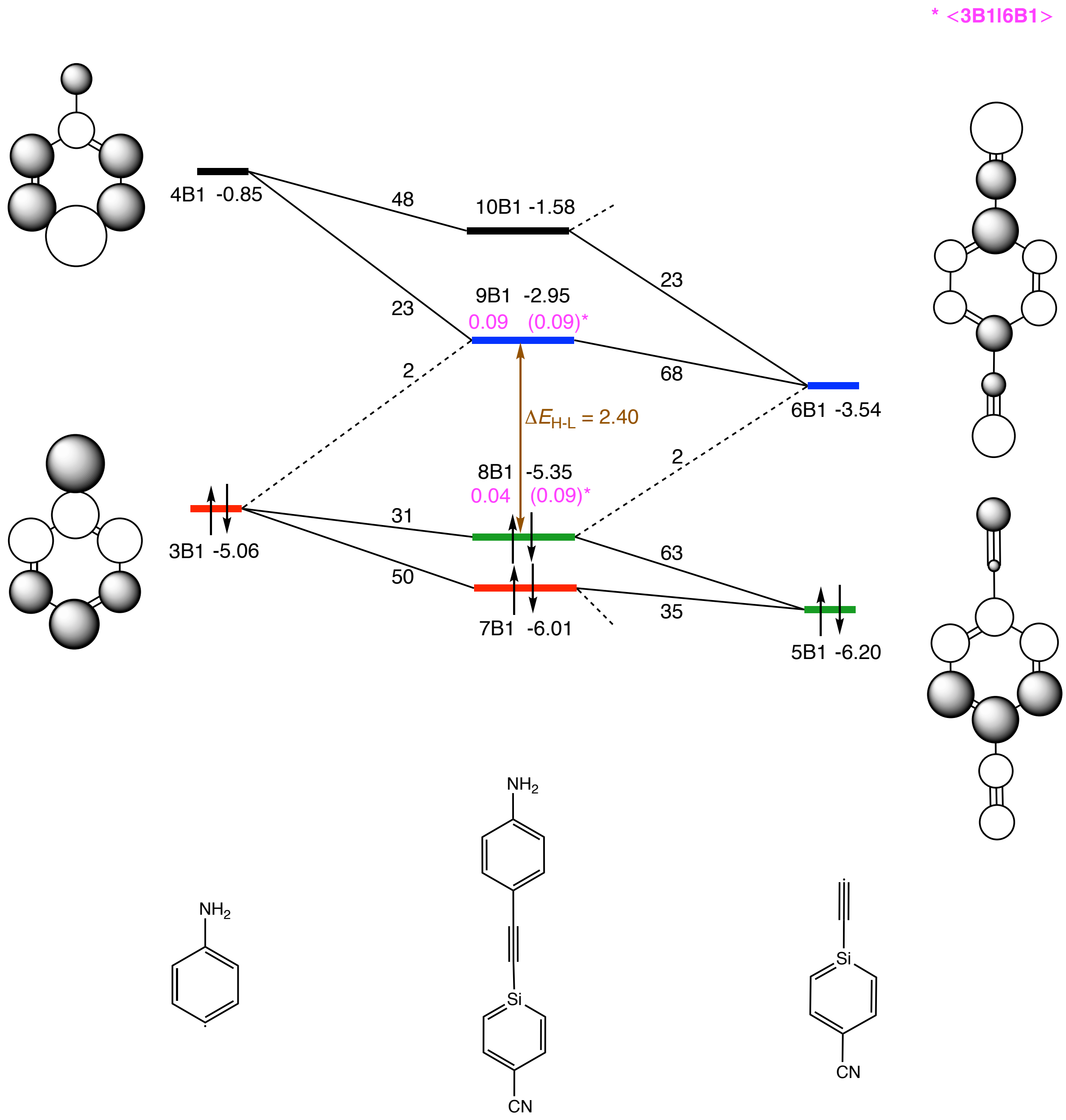

Figure S18. Fragment Frontier Molecular Orbital diagram of D2-π-A2(Si) (see text under Section S1, for details).

Spatial separation between the frontier MO is an indicator for the strength of Intramolecular Charge Transfer (ICT) excitation. With Si in the donor, ICT nature of E_0(S1) is increased if compared to the C analogue.

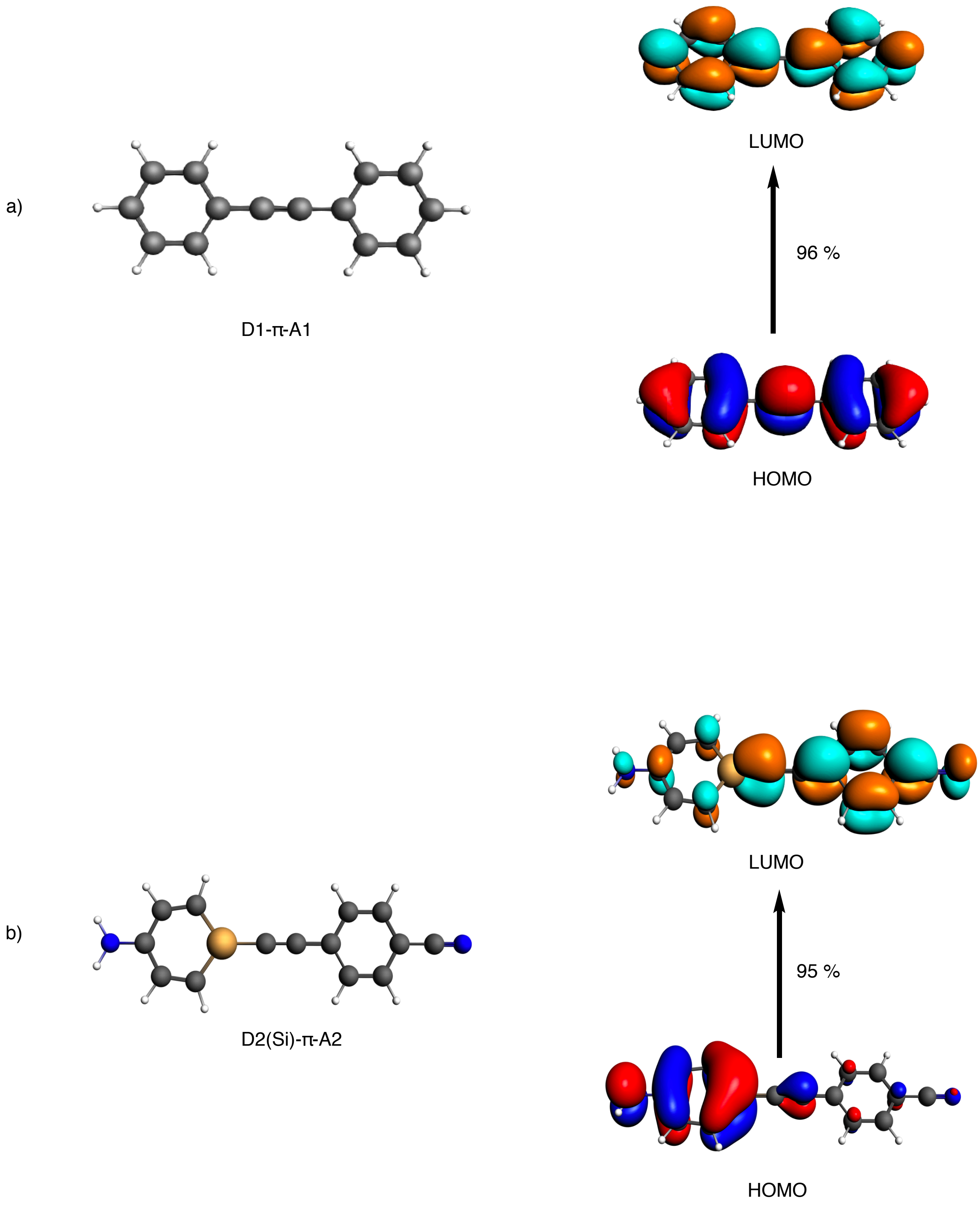

Figure S19. MO density pictures highlighting the spatial separation of frontier MOs (isovalue = 0.03).

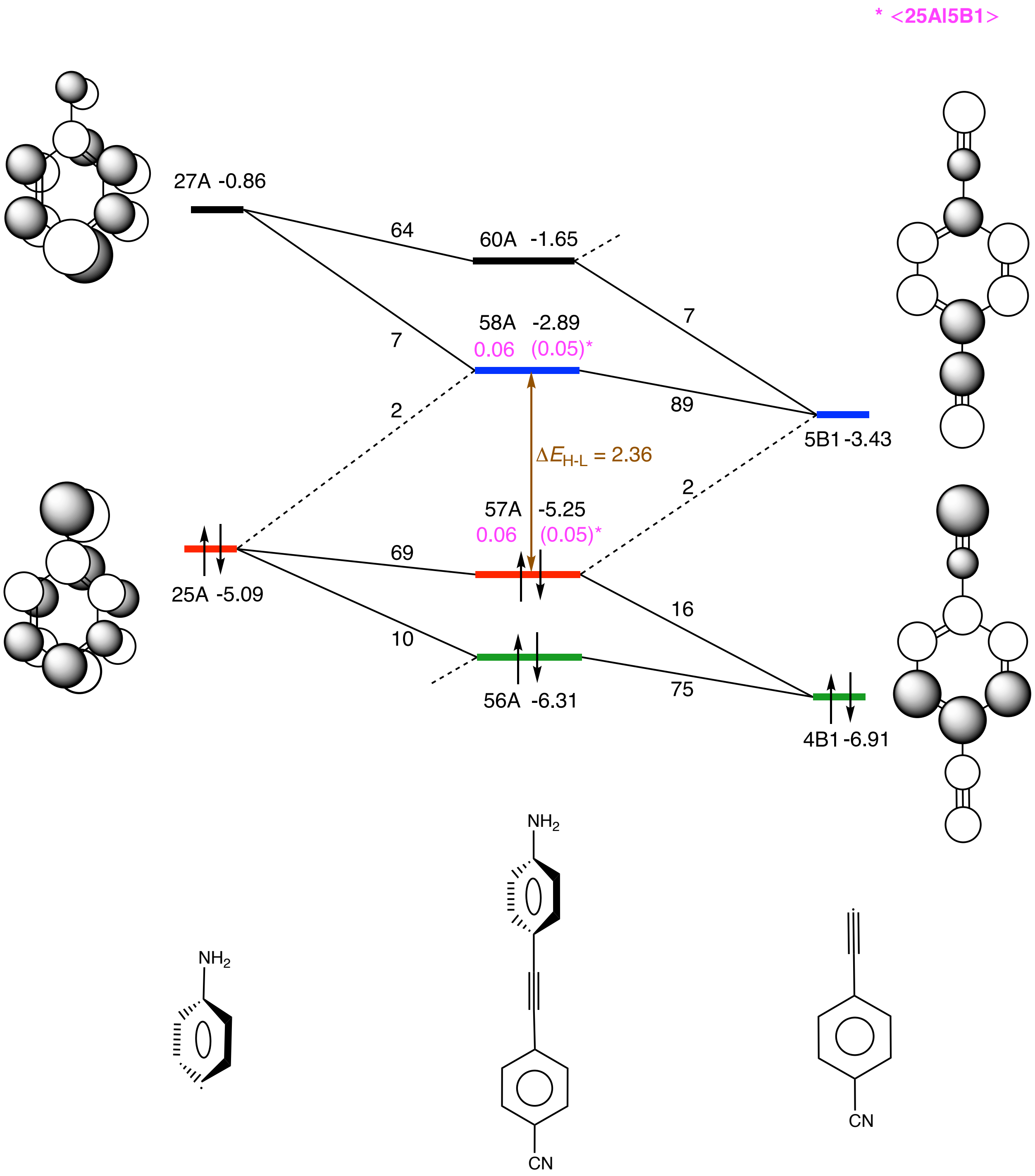

Figure S20. Fragment Frontier Molecular Orbital diagram of D2-π(45°)-A2 (see text under Section S1, for details).

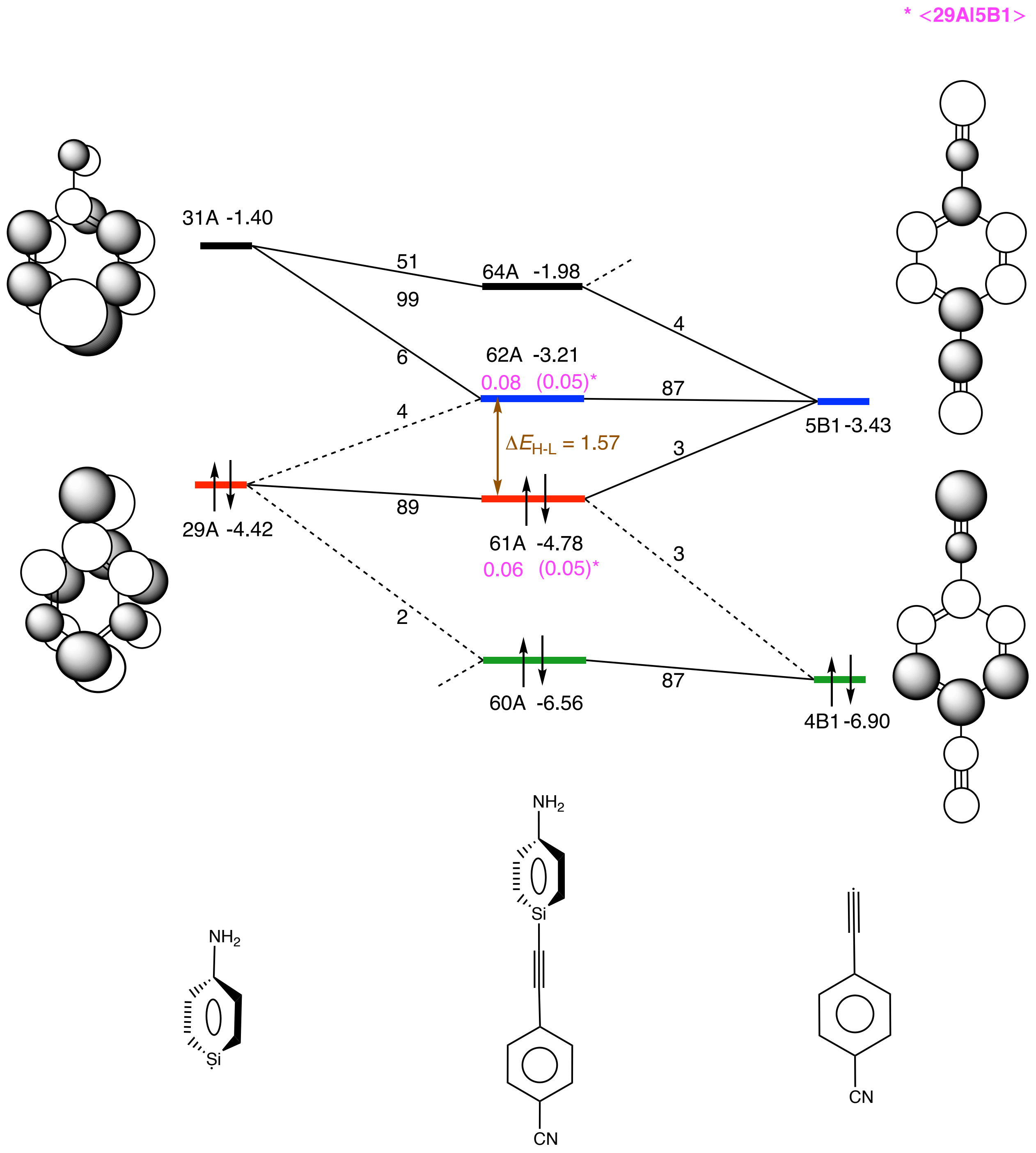

Figure S21. Fragment Frontier Molecular Orbital diagram of D2(Si)-S(45°)-A2 (see text under Section S1, for details).

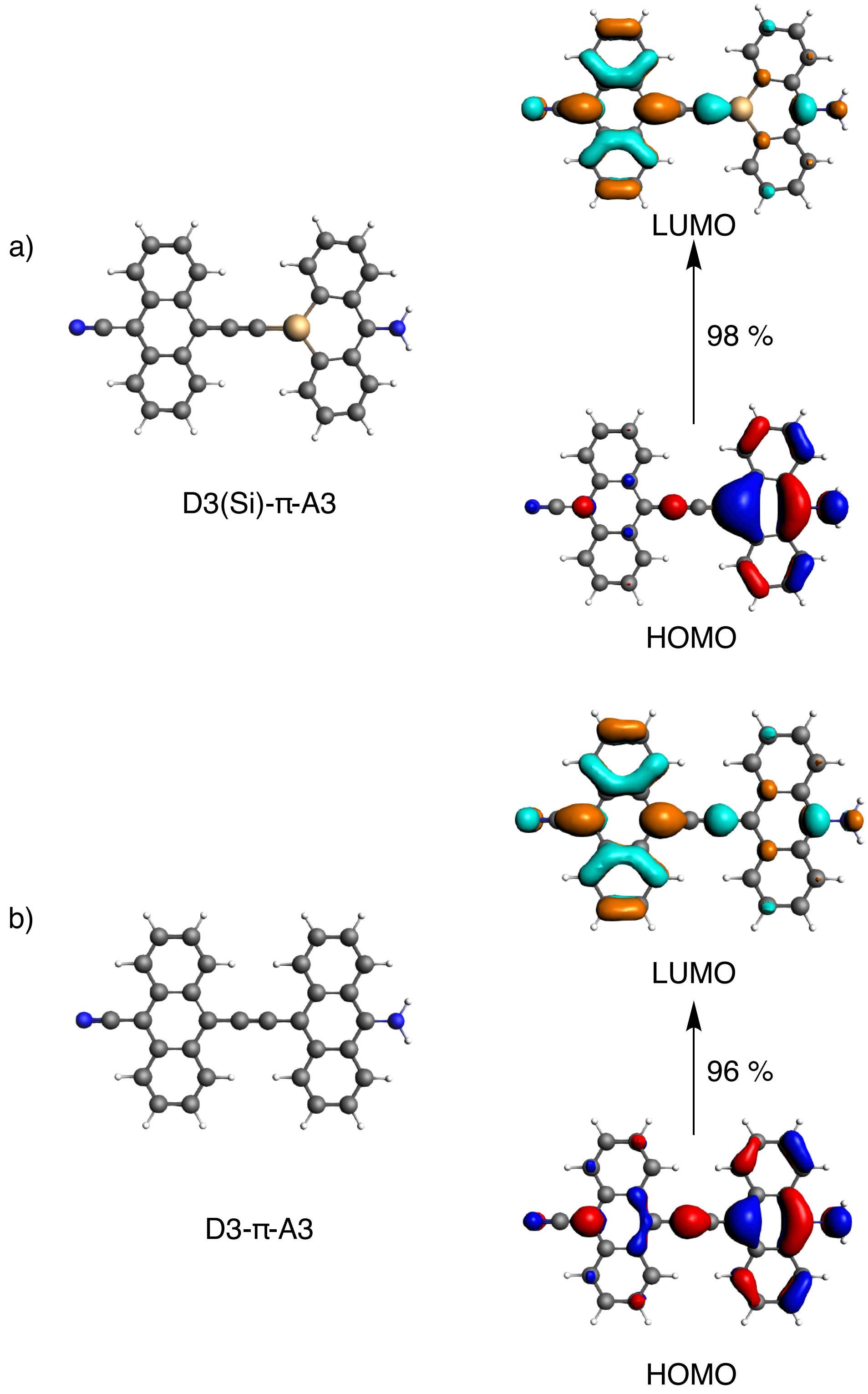

Figure S22. MO density pictures highlighting the spatial separation of frontier MOs (isovalue = 0.03).

S2. References

[1] G. te Velde, F. M. Bickelhaupt, E. J. Baerends, C. Fonseca Guerra, S. J. A. van Gisbergen, J. G. Snijders, T. Ziegler, *J. Comput. Chem.* **2001**, *22*, 931.

[2] C. Fonseca Guerra, J. G. Snijders, G. te Velde, E. J. Baerends, *Theor. Chem. Acc.* **1998**, *99*, 391.

[3] ADF2016, SCM Theoretical Chemistry; Vrije Universiteit, Amsterdam, The Netherlands https://www.scm.com/.

S3. Cartesian coordinates of stationary points, frequencies and total bond energies

D1

ZORA-BP86/TZ2P/COSMO

Total bond energy (Hartree): -2.754

Imaginary frequencies: None

Cartesian coordinates [Å]:

C	1.210768	0.699037	0.000000
C	-1.210768	-0.699037	0.000000
C	0.000000	1.398074	0.000000
C	1.210768	-0.699037	0.000000
C	0.000000	-1.398074	0.000000
C	-1.210768	0.699037	0.000000
H	2.155049	1.244218	0.000000
H	2.155049	-1.244218	0.000000
H	0.000000	-2.488436	0.000000
H	-2.155049	-1.244218	0.000000
H	-2.155049	1.244218	0.000000
H	0.000000	2.488436	0.000000

D1(Si)

ZORA-BP86/TZ2P/COSMO

Total bond energy (Hartree): -2.554

Imaginary frequencies: None

Cartesian coordinates [Å]:

C	0.000000	0.000000	1.059307
Si	0.000000	0.000000	-2.007228
C	0.000000	1.246456	0.411752
C	0.000000	-1.246456	0.411752
C	0.000000	-1.448865	-0.975221
C	0.000000	1.448865	-0.975221
H	0.000000	0.000000	2.149904
H	0.000000	-2.130775	1.056489
H	0.000000	-2.472311	-1.352036
H	0.000000	0.000000	-3.489543
H	0.000000	2.472311	-1.352036
H	0.000000	2.130775	1.056489

D2

ZORA-BP86/TZ2P/COSMO

Total bond energy (Hartree): -2.975

Imaginary frequencies: None

Cartesian coordinates [Å]:

C	0.000000	-1.217169	-0.201936
C	0.000000	1.221029	1.200346
C	0.000000	0.000000	-0.919787
C	0.000000	-1.221029	1.200346

C	0.000000	0.000000	1.838475
C	0.000000	1.217169	-0.201936
N	0.000000	0.000000	-2.295574
H	0.000000	-0.866405	-2.814706
H	0.000000	0.866405	-2.814706
H	0.000000	2.163619	-0.745670
H	0.000000	2.167451	1.742385
H	0.000000	-2.167451	1.742385
H	0.000000	-2.163619	-0.745670

D2(Si)

ZORA-BP86/TZ2P/COSMO

Total bond energy (Hartree): -2.808

Imaginary frequencies: None

Cartesian coordinates [Å]:

C	0.000000	-1.243182	-0.309961
C	0.000000	1.441101	1.074263
C	0.000000	0.000000	-0.985013
C	0.000000	-1.441101	1.074263
Si	0.000000	0.000000	2.146611
C	0.000000	1.243182	-0.309961
N	0.000000	0.000000	-2.371332
H	0.000000	-0.865996	-2.890889
H	0.000000	0.865996	-2.890889
H	0.000000	2.135974	-0.946180
H	0.000000	2.466526	1.444301
H	0.000000	-2.466526	1.444301
H	0.000000	-2.135974	-0.946180

D3

ZORA-BP86/TZ2P/COSMO

Total bond energy (Hartree): -6.116

Imaginary frequencies: None

Cartesian coordinates [Å]:

C	0.000000	-3.687312	-2.045402
C	0.000000	-2.506280	-1.337743
C	0.000000	-2.481264	-4.146522
C	0.000000	-1.240625	-1.990247
C	0.000000	-3.673442	-3.465763
C	0.000000	-1.233809	-3.447466
C	0.000000	0.000000	-1.279047
C	0.000000	1.240625	-1.990247
C	0.000000	2.506280	-1.337743
C	0.000000	1.233809	-3.447466
C	0.000000	0.000000	-4.064636
C	0.000000	3.687312	-2.045402
C	0.000000	3.673442	-3.465763
C	0.000000	2.481264	-4.146522
H	0.000000	-2.563289	-0.249205
H	0.000000	-4.637992	-1.513464

H	0.000000	-4.615094	-4.014524
H	0.000000	-2.460045	-5.236062
H	0.000000	-0.859086	0.617009
H	0.000000	2.460045	-5.236062
H	0.000000	4.615094	-4.014524
H	0.000000	4.637992	-1.513464
H	0.000000	2.563289	-0.249205
N	0.000000	0.000000	0.087974
H	0.000000	0.859086	0.617009

D3(Si)

ZORA-BP86/TZ2P/COSMO

Total bond energy (Hartree): -5.978

Imaginary frequencies: None

Cartesian coordinates [Å]:

C	0.000000	-3.740741	-1.880858
C	0.000000	-2.489434	-1.286516
C	0.000000	-2.707154	-4.048476
C	0.000000	-1.290327	-2.054333
C	0.000000	-3.863104	-3.279631
C	0.000000	-1.410750	-3.483931
C	0.000000	0.000000	-1.380741
C	0.000000	1.290327	-2.054333
C	0.000000	2.489434	-1.286516
C	0.000000	1.410750	-3.483931
Si	0.000000	0.000000	-4.723431
C	0.000000	3.740741	-1.880858
C	0.000000	3.863104	-3.279631
C	0.000000	2.707154	-4.048476
H	0.000000	-2.471806	-0.197477
H	0.000000	-4.631165	-1.252206
H	0.000000	-4.847060	-3.748555
H	0.000000	-2.791987	-5.138809
H	0.000000	-0.857833	0.506206
H	0.000000	2.791987	-5.138809
H	0.000000	4.847060	-3.748555
H	0.000000	4.631165	-1.252206
H	0.000000	2.471806	-0.197477
N	0.000000	0.000000	-0.026256
H	0.000000	0.857833	0.506206

A2

ZORA-BP86/TZ2P/COSMO

Total bond energy (Hartree): -3.004

Imaginary frequencies: None

Cartesian coordinates [Å]:

C	0.000000	0.000000	1.049406
C	0.000000	-1.227206	0.358762
C	0.000000	-1.230412	-1.041292
C	0.000000	0.000000	-1.657544

C	0.000000	1.230412	-1.041292
C	0.000000	1.227206	0.358762
C	0.000000	0.000000	2.476416
N	0.000000	0.000000	3.640853
H	0.000000	-2.164933	0.912314
H	0.000000	-2.169010	-1.594382
H	0.000000	2.169010	-1.594382
H	0.000000	2.164933	0.912314

A2(Si)

ZORA-BP86/TZ2P/COSMO

Total bond energy (Hartree): -2.847

Imaginary frequencies: None

Cartesian coordinates [Å]:

C	0.000000	0.000000	1.103574
C	0.000000	-1.256997	0.449569
C	0.000000	-1.457020	-0.926468
Si	0.000000	0.000000	-1.977793
C	0.000000	1.457020	-0.926468
C	0.000000	1.256997	0.449569
C	0.000000	0.000000	2.526244
N	0.000000	0.000000	3.693453
H	0.000000	-2.139964	1.095026
H	0.000000	-2.477297	-1.307527
H	0.000000	2.477297	-1.307527
H	0.000000	2.139964	1.095026

D2-π

ZORA-BP86/TZ2P/COSMO

Total bond energy (Hartree): -3.569

Imaginary frequencies: None

Cartesian coordinates [Å]:

C	0.000000	-1.226865	-2.543674
C	0.000000	-1.231343	-3.913439
C	0.000000	0.000000	-4.635179
C	0.000000	1.231343	-3.913439
C	0.000000	1.226865	-2.543674
H	0.000000	-2.165588	-1.992944
H	0.000000	-2.170019	-4.466533
H	0.000000	-0.867314	-6.501523
N	0.000000	0.000000	-5.976179
H	0.000000	2.170019	-4.466533
H	0.000000	0.867314	-6.501523
C	0.000000	0.000000	0.851168
C	0.000000	0.000000	-0.411176
H	0.000000	2.165588	-1.992944
C	0.000000	0.000000	-1.804811

D2(Si)-π

ZORA-BP86/TZ2P/COSMO

Total bond energy (Hartree): -3.400

Imaginary frequencies: None

Cartesian coordinates [Å]:

C	0.000000	-1.451453	-2.751758
C	0.000000	-1.270343	-4.112005
C	0.000000	0.000000	-4.780314
C	0.000000	1.270343	-4.112005
C	0.000000	1.451453	-2.751758
H	0.000000	-2.471608	-2.367156
H	0.000000	-2.146408	-4.766967
H	0.000000	-0.867287	-6.654235
N	0.000000	0.000000	-6.129139
H	0.000000	2.146408	-4.766967
H	0.000000	0.867287	-6.654235
C	0.000000	0.000000	1.336627
C	0.000000	0.000000	0.080451
H	0.000000	2.471608	-2.367156
Si	0.000000	0.000000	-1.672870

π-A2

ZORA-BP86/TZ2P/COSMO

Total bond energy (Hartree): -3.565

Imaginary frequencies: None

Cartesian coordinates [Å]:

C	0.000000	0.000000	0.894308
C	0.000000	-1.232084	0.199943
C	0.000000	-1.232786	-1.177890
C	0.000000	0.000000	-1.900234
C	0.000000	1.232786	-1.177890
C	0.000000	1.232084	0.199943
C	0.000000	0.000000	2.317478
N	0.000000	0.000000	3.481742
H	0.000000	-2.165553	0.757344
H	0.000000	-2.168242	-1.732470
H	0.000000	2.168242	-1.732470
H	0.000000	2.165553	0.757344
C	0.000000	0.000000	-3.291017
C	0.000000	0.000000	-4.565765

π-A2(Si)

ZORA-BP86/TZ2P/COSMO

Total bond energy (Hartree): -3.399

Imaginary frequencies: None

Cartesian coordinates [Å]:

C	0.000000	0.000000	1.039190
C	0.000000	-1.278912	0.395719
C	0.000000	-1.466155	-0.964140
Si	0.000000	0.000000	-2.038431
C	0.000000	1.466155	-0.964140
C	0.000000	1.278912	0.395719
C	0.000000	0.000000	2.459004
N	0.000000	0.000000	3.625455
H	0.000000	-2.147240	1.056523
H	0.000000	-2.483656	-1.353841
H	0.000000	2.483656	-1.353841
H	0.000000	2.147240	1.056523
C	0.000000	0.000000	-3.779742
C	0.000000	0.000000	-5.041227

D1-π-A1

ZORA-BP86/TZ2P/COSMO

Total bond energy (Hartree): -5.834

Imaginary frequencies: None

Cartesian coordinates [Å]:

C	0.000000	1.215932	2.749069
C	0.000000	-1.209619	4.141518
C	0.000000	0.000000	2.030421
C	0.000000	1.209619	4.141518
C	0.000000	0.000000	4.843354
C	0.000000	-1.215932	2.749069
H	0.000000	-2.157833	2.202373
H	0.000000	0.000000	5.932983
H	0.000000	-2.155241	4.683220
H	0.000000	2.155241	4.683220
H	0.000000	2.157833	2.202373
C	0.000000	0.000000	0.610020
C	0.000000	0.000000	-0.610020
H	0.000000	2.157833	-2.202373
C	0.000000	0.000000	-2.030421
C	0.000000	-1.215932	-2.749069
C	0.000000	-1.209619	-4.141518
C	0.000000	0.000000	-4.843354
C	0.000000	1.209619	-4.141518
C	0.000000	1.215932	-2.749069
H	0.000000	-2.157833	-2.202373
H	0.000000	-2.155241	-4.683220
H	0.000000	0.000000	-5.932983
H	0.000000	2.155241	-4.683220

D1(Si)-π-A1

ZORA-BP86/TZ2P/COSMO

Total bond energy (Hartree): -5.650

Imaginary frequencies: None

Cartesian coordinates [Å]:

C	0.000000	1.454420	3.135658
C	0.000000	-1.247274	4.520404

Si	0.000000	0.000000	2.111614
C	0.000000	1.247274	4.520404
C	0.000000	0.000000	5.167667
C	0.000000	-1.454420	3.135658
H	0.000000	-2.476768	2.757342
H	0.000000	0.000000	6.258018
H	0.000000	-2.131022	5.165680
H	0.000000	2.131022	5.165680
H	0.000000	2.476768	2.757342
C	0.000000	0.000000	0.321102
C	0.000000	0.000000	-0.905390
H	0.000000	2.159259	-2.488522
C	0.000000	0.000000	-2.322714
C	0.000000	-1.218457	-3.036541
C	0.000000	-1.210832	-4.428236
C	0.000000	0.000000	-5.127935
C	0.000000	1.210832	-4.428236
C	0.000000	1.218457	-3.036541
H	0.000000	-2.159259	-2.488522
H	0.000000	-2.155613	-4.970842
H	0.000000	0.000000	-6.217469
H	0.000000	2.155613	-4.970842

D2-π-A1

ZORA-BP86/TZ2P/COSMO

Total bond energy (Hartree): -6.281

Imaginary frequencies: None

Cartesian coordinates [Å]:

C	-0.00000000	1.21521000	3.28114769
C	-0.00000000	-1.20873000	4.67363769
C	-0.00000000	0.00000000	2.55931769
C	-0.00000000	1.20873000	4.67363769
C	-0.00000000	0.00000000	5.37709769
C	-0.00000000	-1.21521000	3.28114769
H	-0.00000000	-2.15771000	2.73535769
H	-0.00000000	0.00000000	6.46671769
H	-0.00000000	-2.15491000	5.21463769
H	-0.00000000	2.15491000	5.21463769
H	-0.00000000	2.15771000	2.73535769
C	-0.00000000	0.00000000	1.13995769
C	0.00000000	-0.00000000	-0.08212231
H	0.00000000	2.15709000	-1.69105231
C	0.00000000	-0.00000000	-1.49844231
C	0.00000000	-1.21034000	-2.22995231
C	0.00000000	-1.21135000	-3.61620231
C	0.00000000	-0.00000000	-4.34036231
C	0.00000000	1.21135000	-3.61620231
C	0.00000000	1.21034000	-2.22995231
H	-0.00000000	-2.15709000	-1.69105231
H	0.00000000	-2.15863000	-4.15681231
N	0.00000000	-0.00000000	-5.72156231
H	0.00000000	2.15863000	-4.15681231
H	0.00000000	0.89961000	-6.16106231
H	0.00000000	-0.89961000	-6.16106231

D1-π-A2

ZORA-BP86/TZ2P/COSMO

Total bond energy (Hartree): -6.306

Imaginary frequencies: None

Cartesian coordinates [Å]:

C	0.000000	1.217540	2.776058
C	0.000000	-1.219824	4.162016
C	0.000000	0.000000	2.057944
C	0.000000	1.219824	4.162016
C	0.000000	0.000000	4.865730
C	0.000000	-1.217540	2.776058
H	0.000000	-2.159516	2.231228
C	0.000000	0.000000	6.289898
H	0.000000	-2.160318	4.709181
H	0.000000	2.160318	4.709181
H	0.000000	2.159516	2.231228
C	0.000000	0.000000	0.641614
C	0.000000	0.000000	-0.578474
H	0.000000	2.158208	-2.166284
C	0.000000	0.000000	-1.997424
C	0.000000	-1.217114	-2.714080
C	0.000000	-1.210446	-4.106091
C	0.000000	0.000000	-4.806612
C	0.000000	1.210446	-4.106091
C	0.000000	1.217114	-2.714080
H	0.000000	-2.158208	-2.166284
H	0.000000	-2.155379	-4.648678
H	0.000000	0.000000	-5.896211
H	0.000000	2.155379	-4.648678
N	0.000000	0.000000	7.454917

D2-π-A2

ZORA-BP86/TZ2P/COSMO

Total bond energy (Hartree): -6.754

Imaginary frequencies: None

Cartesian coordinates [Å]:

C	0.000000	1.217502	2.971980
C	0.000000	-1.219370	4.356947
C	0.000000	0.000000	2.248538
C	0.000000	1.219370	4.356947
C	0.000000	0.000000	5.064023
C	0.000000	-1.217502	2.971980
H	0.000000	-2.160513	2.428545
C	0.000000	0.000000	6.486341
H	0.000000	-2.160717	4.903088
H	0.000000	2.160717	4.903088
H	0.000000	2.160513	2.428545
C	0.000000	0.000000	0.836368
C	0.000000	0.000000	-0.387000
H	0.000000	2.158530	-1.991187
C	0.000000	0.000000	-1.798206

C	0.000000	-1.212179	-2.530724
C	0.000000	-1.214882	-3.913991
C	0.000000	0.000000	-4.641172
C	0.000000	1.214882	-3.913991
C	0.000000	1.212179	-2.530724
H	0.000000	-2.158530	-1.991187
H	0.000000	-2.161576	-4.455167
N	0.000000	0.000000	-6.004286
H	0.000000	2.161576	-4.455167
H	0.000000	0.865553	-6.526464
H	0.000000	-0.865553	-6.526464
N	0.000000	0.000000	7.652428

D2(Si)-π-A2

ZORA-BP86/TZ2P/COSMO

Total bond energy (Hartree): -6.561

Imaginary frequencies: None

Cartesian coordinates [Å]:

C	0.000000	1.219901	3.301554
C	0.000000	-1.220401	4.686149
C	0.000000	0.000000	2.582736
C	0.000000	1.220401	4.686149
C	0.000000	0.000000	5.391570
C	0.000000	-1.219901	3.301554
H	0.000000	-2.162148	2.757348
C	0.000000	0.000000	6.814285
H	0.000000	-2.161192	5.232872
H	0.000000	2.161192	5.232872
H	0.000000	2.162148	2.757348
C	0.000000	0.000000	1.172685
C	0.000000	0.000000	-0.057173
H	0.000000	2.467719	-2.517430
Si	0.000000	0.000000	-1.839947
C	0.000000	-1.442516	-2.888573
C	0.000000	-1.250616	-4.267244
C	0.000000	0.000000	-4.940109
C	0.000000	1.250616	-4.267244
C	0.000000	1.442516	-2.888573
H	0.000000	-2.467719	-2.517430
H	0.000000	-2.136699	-4.910190
N	0.000000	0.000000	-6.317347
H	0.000000	2.136699	-4.910190
H	0.000000	0.866276	-6.838082
H	0.000000	-0.866276	-6.838082
N	0.000000	0.000000	7.979923

D2-π-(Si)A2

ZORA-BP86/TZ2P/COSMO

Total bond energy (Hartree): -6.576

Imaginary frequencies: None

Cartesian coordinates [Å]:

C	0.000000	1.453138	3.345763
C	0.000000	-1.259135	4.720566
Si	0.000000	0.000000	2.311221
C	0.000000	1.259135	4.720566
C	0.000000	0.000000	5.376846
C	0.000000	-1.453138	3.345763
H	0.000000	-2.475412	2.968452
C	0.000000	0.000000	6.797662
H	0.000000	-2.139216	5.368303
H	0.000000	2.139216	5.368303
H	0.000000	2.475412	2.968452
C	0.000000	0.000000	0.536592
C	0.000000	0.000000	-0.694284
H	0.000000	2.160569	-2.288930
C	0.000000	0.000000	-2.100220
C	0.000000	-1.215142	-2.829478
C	0.000000	-1.216694	-4.210868
C	0.000000	0.000000	-4.937256
C	0.000000	1.216694	-4.210868
C	0.000000	1.215142	-2.829478
H	0.000000	-2.160569	-2.288930
H	0.000000	-2.162608	-4.752924
N	0.000000	0.000000	-6.297203
H	0.000000	2.162608	-4.752924
H	0.000000	0.865759	-6.819680
H	0.000000	-0.865759	-6.819680
N	0.000000	0.000000	7.965985

D2-π(45°)-A2

ZORA-BP86/TZ2P/COSMO

Total bond energy (Hartree): -6.752

Imaginary frequencies: None

Cartesian coordinates [Å]:

C	0.045557	1.231095	2.989431
C	-0.022219	-1.221310	4.344383
C	0.036597	0.023613	2.252704
C	0.030137	1.216596	4.375129
C	-0.004228	-0.011097	5.065112
C	-0.002567	-1.201527	2.958811
H	-0.015629	-2.137112	2.402874
C	-0.024514	-0.028780	6.488218
H	-0.050393	-2.168886	4.878787
H	0.038166	2.150744	4.933282
H	0.065909	2.180286	2.457253
C	0.031140	0.039772	0.836252
C	0.008599	0.047130	-0.385986
H	1.489651	1.584400	-2.026501
C	-0.011377	0.038582	-1.801001
C	-0.843284	-0.856783	-2.513988
C	-0.847533	-0.885626	-3.898202
C	-0.007058	-0.025398	-4.643650
C	0.835761	0.863070	-3.935080
C	0.831400	0.892572	-2.550810
H	-1.495264	-1.531858	-1.960815
H	-1.503093	-1.580594	-4.424020
N	-0.007921	-0.053742	-6.008255

H	1.496479	1.530290	-4.489565
H	0.592502	0.558272	-6.543893
H	-0.608025	-0.688880	-6.516609
N	-0.041163	-0.043289	7.653454

D2(Si)-π(45°)-A2

ZORA-BP86/TZ2P/COSMO

Total bond energy (Hartree): -6.559

Imaginary frequencies: None

Cartesian coordinates [Å]:

C	3.012963	1.344797	1.276105
C	4.135275	-1.062768	2.178897
C	2.315967	0.128005	1.091640
C	4.253722	1.358204	1.892228
C	4.824888	0.153878	2.348288
C	2.896652	-1.074295	1.558440
H	2.363702	-2.013553	1.424868
C	6.100694	0.166853	2.980246
H	4.578970	-1.990652	2.533936
H	4.787170	2.296525	2.029875
H	2.568285	2.274800	0.927764
C	1.044248	0.115469	0.474392
C	-0.063690	0.097377	-0.056928
H	-1.548950	1.665371	-2.793375
Si	-1.682728	0.051325	-0.813313
C	-3.000462	-1.069761	-0.382118
C	-4.159825	-1.048776	-1.153809
C	-4.391222	-0.241203	-2.297923
C	-3.438159	0.677126	-2.810437
C	-2.172462	0.928784	-2.285926
H	-2.969009	-1.749971	0.469522
H	-4.978952	-1.716968	-0.868853
N	-5.602603	-0.356924	-2.946297
H	-3.741596	1.227871	-3.706568
H	-5.809593	0.196677	-3.765849
H	-6.309874	-0.999458	-2.617809
N	7.145547	0.177878	3.495919

D3-π-A3

ZORA-BP86/TZ2P/COSMO

Total bond energy (Hartree): -12.121

Imaginary frequencies: None

Cartesian coordinates [Å]:

C	0.000000	3.660986	4.222725
C	0.000000	2.464396	4.895325
C	0.000000	2.498673	2.090640
C	0.000000	1.222141	4.189791
C	0.000000	3.675691	2.802036
C	0.000000	1.236076	2.745682
C	0.000000	0.000000	4.873238
C	0.000000	-1.222141	4.189791

C	0.000000	-2.464396	4.895325
C	0.000000	-1.236076	2.745682
C	0.000000	0.000000	2.027610
C	0.000000	-3.660986	4.222725
C	0.000000	-3.675691	2.802036
C	0.000000	-2.498673	2.090640
H	0.000000	2.438849	5.985608
H	0.000000	4.601447	4.772967
H	0.000000	4.629242	2.274588
H	0.000000	2.517927	1.002860
C	0.000000	0.000000	0.613155
H	0.000000	-2.517927	1.002860
H	0.000000	-4.629242	2.274588
H	0.000000	-4.601447	4.772967
H	0.000000	-2.438849	5.985608
H	0.000000	-4.629242	-2.274588
C	0.000000	0.000000	-0.613155
C	0.000000	3.675691	-2.802036
C	0.000000	2.498673	-2.090640
C	0.000000	2.464396	-4.895325
C	0.000000	1.236076	-2.745682
C	0.000000	3.660986	-4.222725
C	0.000000	1.222141	-4.189791
C	0.000000	0.000000	-2.027610
C	0.000000	-1.236076	-2.745682
C	0.000000	-2.498673	-2.090640
C	0.000000	-1.222141	-4.189791
C	0.000000	0.000000	-4.873238
C	0.000000	-3.675691	-2.802036
C	0.000000	-3.660986	-4.222725
C	0.000000	-2.464396	-4.895325
H	0.000000	2.517927	-1.002860
H	0.000000	4.629242	-2.274588
H	0.000000	4.601447	-4.772967
H	0.000000	2.438849	-5.985608
H	0.000000	-2.517927	-1.002860
H	0.000000	-2.438849	-5.985608
H	0.000000	-4.601447	-4.772967
H	0.000000	0.000000	5.964501
H	0.000000	0.000000	-5.964501

D4-π-A4

ZORA-BP86/TZ2P/COSMO

Total bond energy (Hartree): -13.038

Imaginary frequencies: None

Cartesian coordinates [Å]:

C	-2.434910	-2.771843	0.000000
C	-1.745027	-1.580322	0.000000
C	-4.529495	-1.566178	0.000000
C	-2.418368	-0.326828	0.000000
C	-3.848590	-2.761812	0.000000
C	-3.854552	-0.315511	0.000000
C	-1.694832	0.910923	0.000000
C	-2.409231	2.154028	0.000000
C	-1.726851	3.402658	0.000000
C	-3.845462	2.153151	0.000000
C	-4.578805	0.921513	0.000000

C	-2.408121	4.599123	0.000000
C	-3.821832	4.599361	0.000000
C	-4.511346	3.408693	0.000000
H	-0.656862	-1.623246	0.000000
H	-1.893587	-3.716846	0.000000
H	-4.398115	-3.702681	0.000000
H	-5.616941	-1.559270	0.000000
C	-5.981607	0.926680	0.000000
H	-5.598833	3.409738	0.000000
H	-4.364519	5.544190	0.000000
H	-1.859971	5.540178	0.000000
H	-0.638407	3.437866	0.000000
N	-0.344471	0.905753	0.000000
C	-7.212691	0.927765	0.000000
C	-9.372646	-2.745441	0.000000
C	-8.678051	-1.558328	0.000000
C	-11.473503	-1.543524	0.000000
C	-9.344847	-0.303800	0.000000
C	-10.789244	-2.736182	0.000000
C	-10.782720	-0.298190	0.000000
C	-8.617294	0.932888	0.000000
C	-9.335973	2.174782	0.000000
C	-8.660343	3.424534	0.000000
C	-10.773866	2.179336	0.000000
C	-11.475119	0.943054	0.000000
C	-9.346546	4.616536	0.000000
C	-10.763167	4.617340	0.000000
C	-11.455818	3.429551	0.000000
H	-7.590542	-1.560743	0.000000
H	-8.836691	-3.693759	0.000000
H	-11.337469	-3.677738	0.000000
H	-12.563161	-1.539050	0.000000
C	-12.891210	0.948153	0.000000
H	-12.545481	3.432776	0.000000
H	-11.304727	5.562749	0.000000
H	-8.803880	5.561026	0.000000
H	-7.572871	3.419174	0.000000
N	-14.059718	0.952348	0.000000
H	0.183386	0.044671	0.000000
H	0.189943	1.762741	0.000000

D4(Si)-π-A4

ZORA-BP86/TZ2P/COSMO

Total bond energy (Hartree): -12.844

Imaginary frequencies: None

Cartesian coordinates [Å]:

C	-1.889749	-2.834157	0.000000
C	-1.314410	-1.581145	0.000000
C	-4.083787	-1.851413	0.000000
C	-2.090142	-0.380219	0.000000
C	-3.293705	-2.981344	0.000000
C	-3.524468	-0.548501	0.000000
C	-1.425623	0.910010	0.000000
C	-2.083077	2.203832	0.000000
C	-1.300802	3.400528	0.000000
C	-3.516490	2.379893	0.000000
Si	-4.561124	0.918535	0.000000
C	-1.869347	4.656623	0.000000

C	-3.272495	4.811461	0.000000
C	-4.068719	3.685859	0.000000
H	-0.226998	-1.549744	0.000000
H	-1.249775	-3.715997	0.000000
H	-3.743221	-3.973672	0.000000
H	-5.169746	-1.952428	0.000000
C	-6.323024	0.923867	0.000000
H	-5.154117	3.792860	0.000000
H	-3.716591	5.806229	0.000000
H	-1.224590	5.534977	0.000000
H	-0.213557	3.363323	0.000000
N	-0.062869	0.906305	0.000000
C	-7.561006	0.927360	0.000000
C	-9.694745	-2.751176	0.000000
C	-8.999632	-1.561938	0.000000
C	-11.797744	-1.551011	0.000000
C	-9.673680	-0.312442	0.000000
C	-11.110004	-2.743551	0.000000
C	-11.109934	-0.305292	0.000000
C	-8.954592	0.931338	0.000000
C	-9.666255	2.179323	0.000000
C	-8.984640	3.424660	0.000000
C	-11.102507	2.180748	0.000000
C	-11.802191	0.939802	0.000000
C	-9.672593	4.618059	0.000000
C	-11.087869	4.618975	0.000000
C	-11.782799	3.430594	0.000000
H	-7.910516	-1.563536	0.000000
H	-9.157245	-3.698710	0.000000
H	-11.657090	-3.685804	0.000000
H	-12.887467	-1.549521	0.000000
C	-13.216374	0.944037	0.000000
H	-12.872510	3.435652	0.000000
H	-11.629266	5.564512	0.000000
H	-9.129345	5.562303	0.000000
H	-7.895537	3.419726	0.000000
N	-14.385547	0.947557	0.000000
H	0.467893	0.048872	0.000000
H	0.472321	1.760949	0.000000

D1-π-A1(N)

ZORA-BP86/TZ2P/COSMO

Total bond energy (Hartree): -5.665

Imaginary frequencies: None

Cartesian coordinates [Å]:

C	0.000000	0.000000	-4.865232
C	0.000000	0.000000	-2.056764
C	0.000000	1.210503	-4.164903
C	0.000000	-1.210503	-4.164903
C	0.000000	-1.217093	-2.772875
C	0.000000	1.217093	-2.772875
H	0.000000	0.000000	-5.954817
H	0.000000	-2.155375	-4.707575
H	0.000000	-2.157867	-2.224570
C	0.000000	0.000000	-0.637344
H	0.000000	2.157867	-2.224570
H	0.000000	2.155375	-4.707575
C	0.000000	0.000000	0.582113

H	0.000000	-2.162985	2.219112
C	0.000000	0.000000	1.999312
C	0.000000	1.203320	2.732337
C	0.000000	1.146417	4.121547
N	0.000000	0.000000	4.826978
C	0.000000	-1.146417	4.121547
C	0.000000	-1.203320	2.732337
H	0.000000	2.162985	2.219112
H	0.000000	2.070463	4.702917
H	0.000000	-2.070463	4.702917

D1-π-A1(P)

ZORA-BP86/TZ2P/COSMO

Total bond energy (Hartree): -5.531

Imaginary frequencies: None

Cartesian coordinates [Å]:

P	0.000000	0.000000	-5.246075
C	0.000000	0.000000	-2.041959
C	0.000000	-1.336773	-4.118090
C	0.000000	1.336773	-4.118090
C	0.000000	1.233061	-2.735375
C	0.000000	-1.233061	-2.735375
H	0.000000	-2.145107	-2.135777
H	0.000000	-2.338948	-4.552264
C	0.000000	0.000000	-0.624269
H	0.000000	2.145107	-2.135777
H	0.000000	2.338948	-4.552264
C	0.000000	0.000000	0.596307
H	0.000000	-2.158161	2.186024
C	0.000000	0.000000	2.015282
C	0.000000	1.216591	2.733220
C	0.000000	1.210049	4.125345
C	0.000000	0.000000	4.826641
C	0.000000	-1.210049	4.125345
C	0.000000	-1.216591	2.733220
H	0.000000	2.158161	2.186024
H	0.000000	2.155394	4.667366
H	0.000000	0.000000	5.916221
H	0.000000	-2.155394	4.667366

D1(Ge)-π-A1

ZORA-BP86/TZ2P/COSMO

Total bond energy (Hartree): -5.585

Imaginary frequencies: None

Cartesian coordinates [Å]:

C	0.000000	1.495043	3.211517
C	0.000000	-1.253865	4.585728
Ge	0.000000	0.000000	2.126482
C	0.000000	1.253865	4.585728
C	0.000000	0.000000	5.221629
C	0.000000	-1.495043	3.211517
H	0.000000	-2.515749	2.833561
H	0.000000	0.000000	6.311935

H	0.000000	-2.128684	5.243562
H	0.000000	2.128684	5.243562
H	0.000000	2.515749	2.833561
C	0.000000	0.000000	0.266858
C	0.000000	0.000000	-0.957388
H	0.000000	2.158680	-2.543329
C	0.000000	0.000000	-2.376939
C	0.000000	-1.217761	-3.091237
C	0.000000	-1.210578	-4.483264
C	0.000000	0.000000	-5.183272
C	0.000000	1.210578	-4.483264
C	0.000000	1.217761	-3.091237
H	0.000000	-2.158680	-2.543329
H	0.000000	-2.155493	-5.025719
H	0.000000	0.000000	-6.272827
H	0.000000	2.155493	-5.025719

D1(Sn)-*π*-A1

ZORA-BP86/TZ2P/COSMO

Total bond energy (Hartree): -5.520

Imaginary frequencies: None

Cartesian coordinates [Å]:

C	0.000000	1.588060	3.435625
C	0.000000	-1.268305	4.791651
Sn	0.000000	0.000000	2.148966
C	0.000000	1.268305	4.791651
C	0.000000	0.000000	5.404523
C	0.000000	-1.588060	3.435625
H	0.000000	-2.630179	3.121279
H	0.000000	0.000000	6.495236
H	0.000000	-2.117019	5.485654
H	0.000000	2.117019	5.485654
H	0.000000	2.630179	3.121279
C	0.000000	0.000000	0.099204
C	0.000000	0.000000	-1.125624
H	0.000000	2.158357	-2.713602
C	0.000000	0.000000	-2.546860
C	0.000000	-1.217312	-3.261427
C	0.000000	-1.210427	-4.653627
C	0.000000	0.000000	-5.353808
C	0.000000	1.210427	-4.653627
C	0.000000	1.217312	-3.261427
H	0.000000	-2.158357	-2.713602
H	0.000000	-2.155455	-5.195955
H	0.000000	0.000000	-6.443383
H	0.000000	2.155455	-5.195955

D4-*π*-A3(N)

ZORA-BP86/TZ2P/COSMO

Total bond energy (Hartree): -12.401

Imaginary frequencies: None

Cartesian coordinates [Å]:

C	0.000000	3.685534	3.796389

C	0.000000	2.492172	4.483018
C	0.000000	2.486740	1.697547
C	0.000000	1.240286	3.806289
C	0.000000	3.679966	2.382301
C	0.000000	1.234013	2.369828
C	0.000000	0.000000	4.525294
C	0.000000	-1.240286	3.806289
C	0.000000	-2.492172	4.483018
C	0.000000	-1.234013	2.369828
C	0.000000	0.000000	1.642686
C	0.000000	-3.685534	3.796389
C	0.000000	-3.679966	2.382301
C	0.000000	-2.486740	1.697547
H	0.000000	2.532139	5.571275
H	0.000000	4.629048	4.340386
H	0.000000	4.622714	1.835880
H	0.000000	2.481971	0.609688
C	0.000000	0.000000	0.239411
H	0.000000	-2.481971	0.609688
H	0.000000	-4.622714	1.835880
H	0.000000	-4.629048	4.340386
H	0.000000	-2.532139	5.571275
N	0.000000	0.000000	5.876534
C	0.000000	0.000000	-0.990453
C	0.000000	3.657749	-3.254578
C	0.000000	2.499450	-2.512038
C	0.000000	2.380605	-5.313934
C	0.000000	1.223308	-3.134985
C	0.000000	3.595789	-4.673059
C	0.000000	1.157740	-4.576415
C	0.000000	0.000000	-2.395635
C	0.000000	-1.223308	-3.134985
C	0.000000	-2.499450	-2.512038
C	0.000000	-1.157740	-4.576415
N	0.000000	0.000000	-5.271861
C	0.000000	-3.657749	-3.254578
C	0.000000	-3.595789	-4.673059
C	0.000000	-2.380605	-5.313934
H	0.000000	2.544666	-1.424401
H	0.000000	4.626743	-2.756537
H	0.000000	4.518892	-5.252481
H	0.000000	2.311164	-6.401722
H	0.000000	0.859229	6.407346
H	0.000000	-2.311164	-6.401722
H	0.000000	-4.518892	-5.252481
H	0.000000	-4.626743	-2.756537
H	0.000000	-2.544666	-1.424401
H	0.000000	-0.859229	6.407346

D4-π-A3(P)

ZORA-BP86/TZ2P/COSMO

Total bond energy (Hartree): -12.261

Imaginary frequencies: None

Cartesian coordinates [Å]:

C	0.000000	3.684554	3.768574
C	0.000000	2.490201	4.452921
C	0.000000	2.487222	1.669576
C	0.000000	1.238268	3.775400

C	0.000000	3.680189	2.354405
C	0.000000	1.232163	2.337653
C	0.000000	0.000000	4.495047
C	0.000000	-1.238268	3.775400
C	0.000000	-2.490201	4.452921
C	0.000000	-1.232163	2.337653
C	0.000000	0.000000	1.606806
C	0.000000	-3.684554	3.768574
C	0.000000	-3.680189	2.354405
C	0.000000	-2.487222	1.669576
H	0.000000	2.528553	5.541249
H	0.000000	4.627372	4.313850
H	0.000000	4.623184	1.808424
H	0.000000	2.485512	0.582471
C	0.000000	0.000000	0.199521
H	0.000000	-2.485512	0.582471
H	0.000000	-4.623184	1.808424
H	0.000000	-4.627372	4.313850
H	0.000000	-2.528553	5.541249
N	0.000000	0.000000	5.848290
C	0.000000	0.000000	-1.031565
C	0.000000	3.711699	-3.009709
C	0.000000	2.480157	-2.397324
C	0.000000	2.653566	-5.175052
C	0.000000	1.261577	-3.136255
C	0.000000	3.804582	-4.422788
C	0.000000	1.359749	-4.572548
C	0.000000	0.000000	-2.442587
C	0.000000	-1.261577	-3.136255
C	0.000000	-2.480157	-2.397324
C	0.000000	-1.359749	-4.572548
P	0.000000	0.000000	-5.687172
C	0.000000	-3.711699	-3.009709
C	0.000000	-3.804582	-4.422788
C	0.000000	-2.653566	-5.175052
H	0.000000	2.417145	-1.312234
H	0.000000	4.619099	-2.406356
H	0.000000	4.780658	-4.907258
H	0.000000	2.713392	-6.265166
H	0.000000	0.859133	6.378914
H	0.000000	-2.713392	-6.265166
H	0.000000	-4.780658	-4.907258
H	0.000000	-4.619099	-2.406356
H	0.000000	-2.417145	-1.312234
H	0.000000	-0.859133	6.378914

D4(Ge)-π-A4

ZORA-BP86/TZ2P/COSMO

Total bond energy (Hartree): -12.779

Imaginary frequencies: None

Cartesian coordinates [Å]:

C	-1.801376	-2.843036	0.000000
C	-1.243604	-1.582438	0.000000
C	-4.008070	-1.891493	0.000000
C	-2.033102	-0.388002	0.000000
C	-3.202678	-3.011011	0.000000
C	-3.459688	-0.588039	0.000000
C	-1.377170	0.909095	0.000000
C	-2.024647	2.210424	0.000000

C	-1.227338	3.399680	0.000000
C	-3.449910	2.419748	0.000000
Ge	-4.554994	0.919419	0.000000
C	-1.776894	4.663875	0.000000
C	-3.177072	4.841001	0.000000
C	-3.989781	3.726765	0.000000
H	-0.156879	-1.540035	0.000000
H	-1.148407	-3.715230	0.000000
H	-3.638923	-4.009195	0.000000
H	-5.092622	-2.004071	0.000000
C	-6.383839	0.925052	0.000000
H	-5.073570	3.846430	0.000000
H	-3.606767	5.842023	0.000000
H	-1.118250	5.531795	0.000000
H	-0.140884	3.350255	0.000000
N	-0.013805	0.904762	0.000000
C	-7.618159	0.928449	0.000000
C	-9.752166	-2.749506	0.000000
C	-9.057790	-1.560270	0.000000
C	-11.856680	-1.551322	0.000000
C	-9.732930	-0.310707	0.000000
C	-11.167821	-2.742966	0.000000
C	-11.169649	-0.305013	0.000000
C	-9.015066	0.932085	0.000000
C	-9.726571	2.178522	0.000000
C	-9.045062	3.424614	0.000000
C	-11.163302	2.180154	0.000000
C	-11.862238	0.939352	0.000000
C	-9.733388	4.617376	0.000000
C	-11.149060	4.618065	0.000000
C	-11.843975	3.429953	0.000000
H	-7.968730	-1.560192	0.000000
H	-9.214134	-3.696762	0.000000
H	-11.713927	-3.685797	0.000000
H	-12.946399	-1.550752	0.000000
C	-13.276955	0.942999	0.000000
H	-12.933683	3.434925	0.000000
H	-11.690360	5.563665	0.000000
H	-9.190530	5.561869	0.000000
H	-7.956015	3.419049	0.000000
N	-14.445937	0.946028	0.000000
H	0.516571	0.047463	0.000000
H	0.521845	1.758751	0.000000

D4(Sn)-π-A4

ZORA-BP86/TZ2P/COSMO

Total bond energy (Hartree): -12.709

Imaginary frequencies: None

Cartesian coordinates [Å]:

C	-1.508837	-2.861619	0.000000
C	-1.005260	-1.578117	0.000000
C	-3.746050	-2.000085	0.000000
C	-1.837057	-0.407585	0.000000
C	-2.898734	-3.090660	0.000000
C	-3.252393	-0.676505	0.000000
C	-1.203275	0.908398	0.000000
C	-1.828167	2.228576	0.000000
C	-0.988411	3.393412	0.000000

C	-3.241652	2.507073	0.000000
Sn	-4.559007	0.919745	0.000000
C	-1.483246	4.680292	0.000000
C	-2.871569	4.918755	0.000000
C	-3.726289	3.833975	0.000000
H	0.079168	-1.504535	0.000000
H	-0.816562	-3.703178	0.000000
H	-3.295595	-4.105147	0.000000
H	-4.824242	-2.162464	0.000000
C	-6.574656	0.925805	0.000000
H	-4.803361	4.003605	0.000000
H	-3.261489	5.935928	0.000000
H	-0.785297	5.517144	0.000000
H	0.095492	3.312360	0.000000
N	0.163653	0.903937	0.000000
C	-7.808635	0.928744	0.000000
C	-9.943274	-2.749733	0.000000
C	-9.248713	-1.560138	0.000000
C	-12.047704	-1.551463	0.000000
C	-9.924160	-0.310690	0.000000
C	-11.358633	-2.743456	0.000000
C	-11.361013	-0.305277	0.000000
C	-9.207056	0.932321	0.000000
C	-9.917764	2.179015	0.000000
C	-9.235915	3.424971	0.000000
C	-11.354616	2.181001	0.000000
C	-12.053632	0.939659	0.000000
C	-9.924352	4.618133	0.000000
C	-11.339723	4.619135	0.000000
C	-12.034896	3.430702	0.000000
H	-8.159528	-1.560031	0.000000
H	-9.404970	-3.696900	0.000000
H	-11.904825	-3.686263	0.000000
H	-13.137454	-1.551152	0.000000
C	-13.468005	0.943336	0.000000
H	-13.124634	3.435977	0.000000
H	-11.881068	5.564735	0.000000
H	-9.381153	5.562497	0.000000
H	-8.146745	3.419258	0.000000
N	-14.637156	0.946390	0.000000
H	0.695036	0.048316	0.000000
H	0.700709	1.755974	0.000000
